# Supplementary material for: Optimization of siRNA therapeutics targeting MIAT for cardioprotection in myocardial ischemia/reperfusion injury
Source: Mol Ther Nucleic Acids. 2025 Oct 18;36(4):102747. doi: 10.1016/j.omtn.2025.102747 (PMC12617760; doi:10.1016/j.omtn.2025.102747)
Supplement: Document S1. Figures S1–S5 and Tables S1 and S2 [file mmc1.pdf]

OMTN, Volume *36*

Supplemental information

Optimization of siRNA therapeutics targeting MIAT for cardioprotection in myocardial ischemia/reperfusion injury

Xiao-Rong Ma, Tong-Meng Yan, Yu Pan, and Zhi-Hong Jiang

Supplemental information

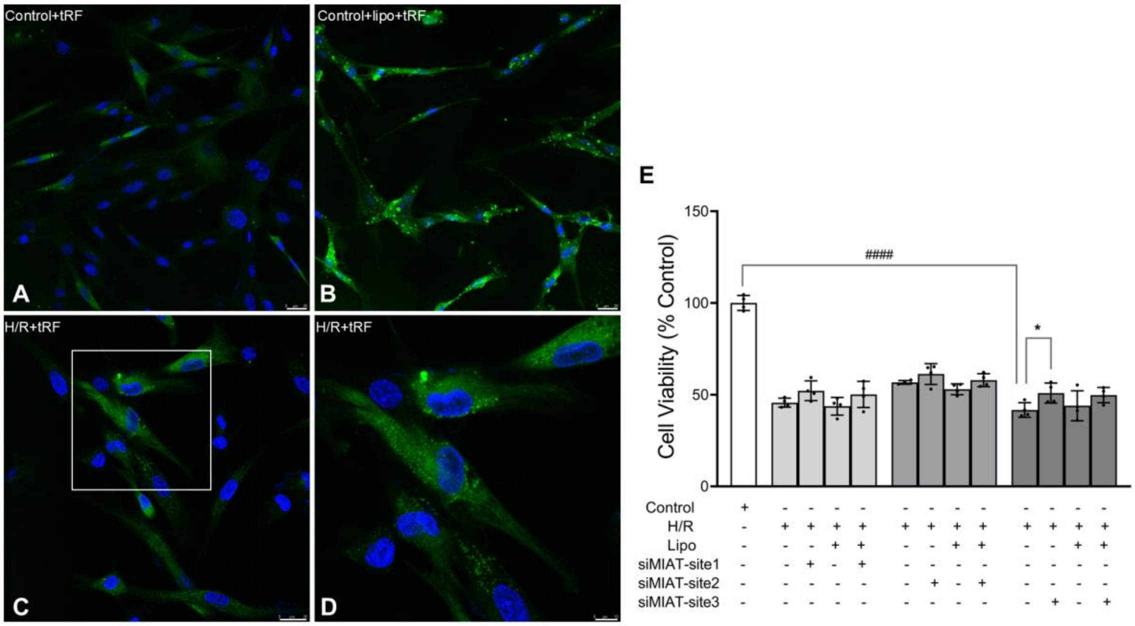

Figure S1. tRF efficiently entered H/R-injured cells without the use of transfection reagents.

Nuclei were stained with Hoechst 33342, appearing blue. (A) The image shows normal cells treated with 100 nM Cy5-labeled HC83 without a transfection reagent. (B) The fluorescence image shows normal cells treated with 100 nM HC83, utilizing Lipofectamine RNAiMAX as the transfection reagent. (C) The image of H/R-injured cells treated with 100 nM Cy5-labeled HC83 without using transfection reagents. Images (A) and (B) were taken with a 20 × objective lens, with scale bars at 25 μm. (D) Offers a magnified view of the area marked in (C), with a 10 μm scale bar, emphasizing the enhanced cellular uptake of HC83 in H/R-injured cells. (E) The comparison of the effects of siMIAT-site1, siMIAT-site2, and siMIAT-site3 on the viability of H/R-induced injured cells at a concentration of 100 nM, both with or without Lipofectamine, reveals that siRNAs demonstrate slightly greater protective activity when the transfection reagent is not used. Data are presented as means ± SDs, n=4. P values were calculated using One-way ANOVA. ####$p < 0.0001$ vs. Control; ∗$p < 0.05$ vs. H/R.

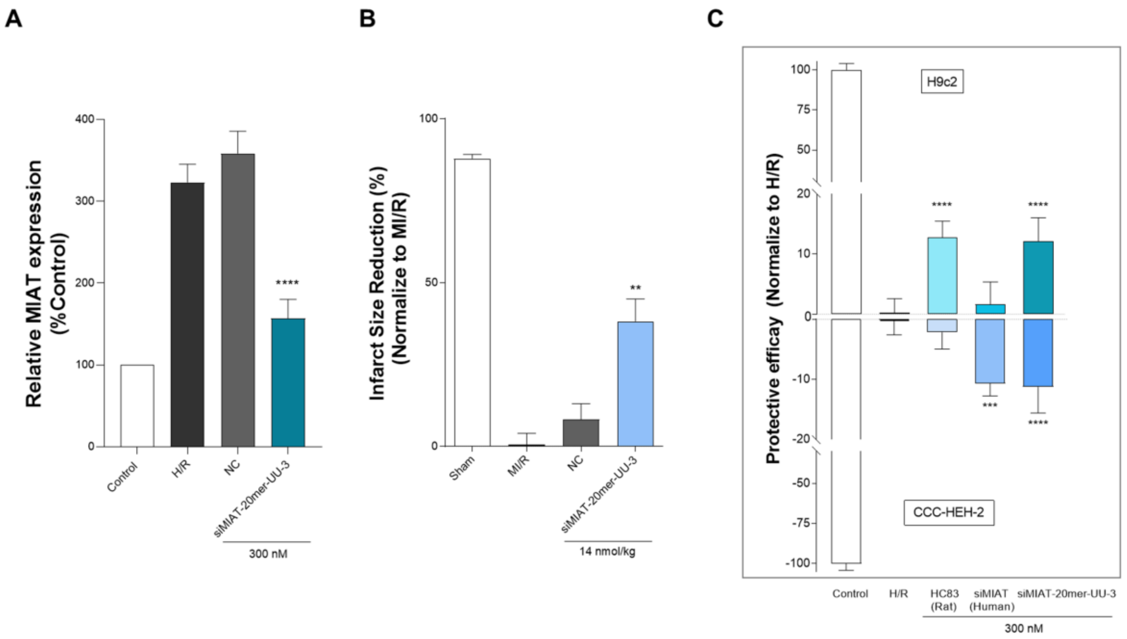

Figure S2. Effects of siRNA treatments on MIAT expression and cardiomyocytes protection.

(A) MIAT expression in H9c2 cardiomyocytes under H/R injury treated with siNC or siMIAT-20mer-UU-3. (B) Infarct size in MI/R model rats treated with siNC or siMIAT-20mer-UU-3 (14 ng/kg). (C) Cell viability in H9c2 and CCC-HEH-2 cardiomyocytes treated with: HC83 (rat-specific), siMIAT-human (human-specific), or siMIAT-20mer-UU-3. Data are presented as means ± SDs. P values were calculated using One-way ANOVA. **p < 0.01 vs. MI/R, ***p < 0.001, ****p<0.0001 vs. H/R.

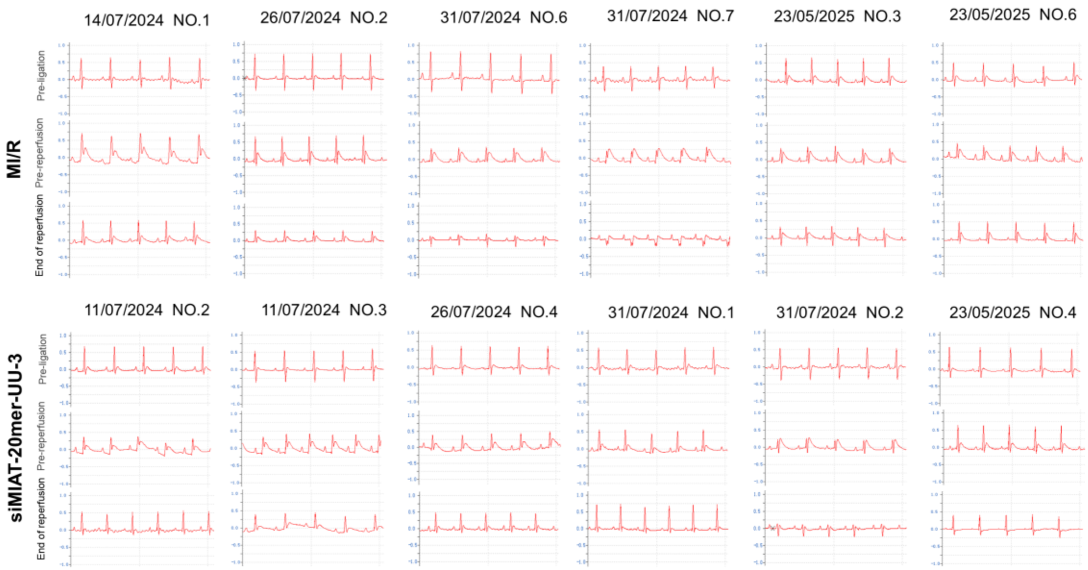

Figure S3. ECG recordings from MI/R and siMIAT-20mer-UU-3 treatment groups.

Comparison of ECG recordings between MI/R and siMIAT-20mer-UU-3 treatment groups across different time points.

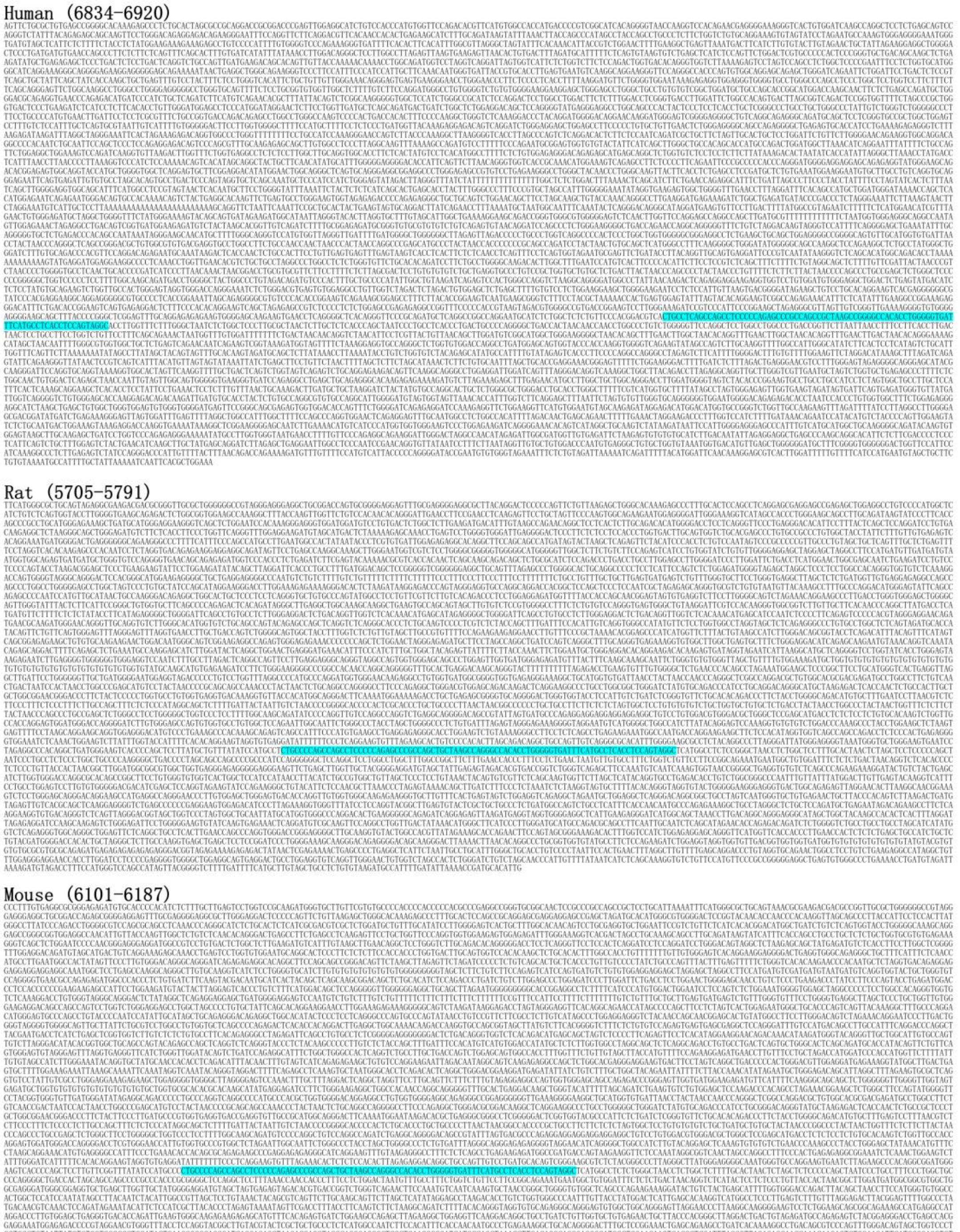

Figure S4. The complete FASTA sequences and homologous regions of lncRNA MIAT from three species.

The blue-highlighted portions indicate the homologous regions of the three sequences.

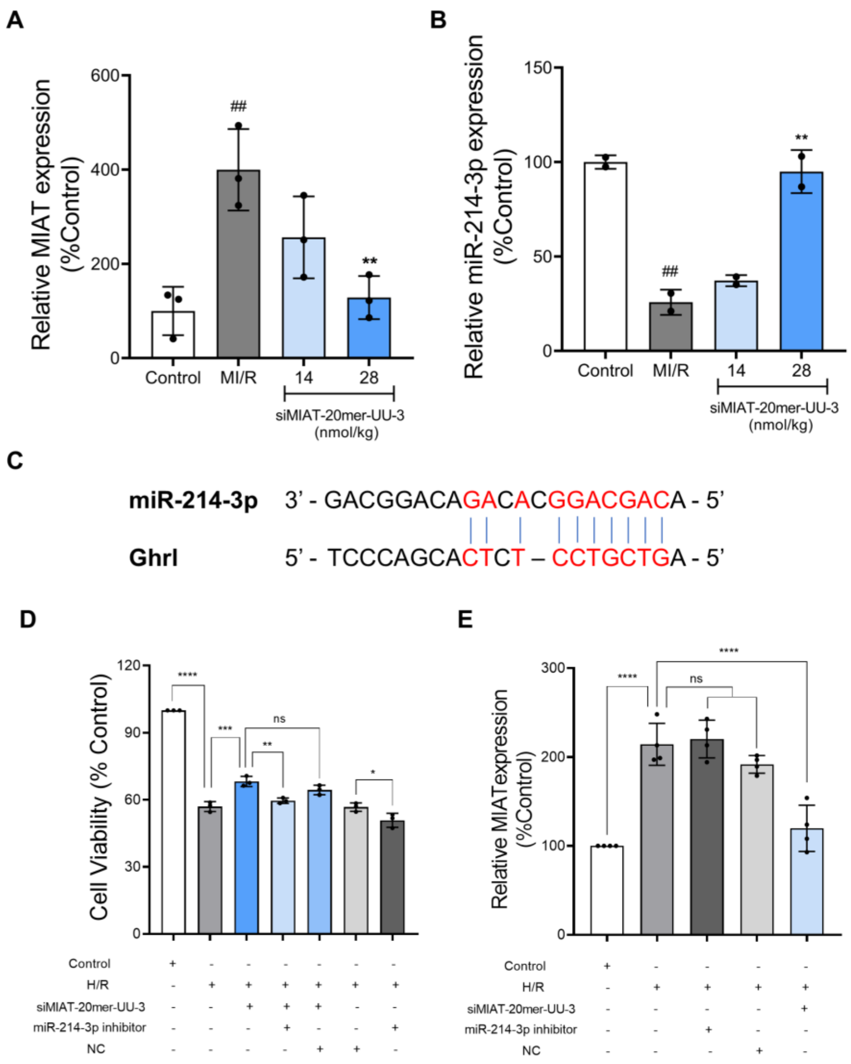

Figure S5. siMIAT-20mer-UU-3 treatment reversed MIAT upregulation and affected miR-214-3p expression, which may potentially target Ghrl.

(A) Relative MIAT expression levels (% of control) were measured in different groups: control, MI/R, and siMIAT-20mer-UU-3 treated at 14 and 28 nmol/kg dosages. (B) Relative miR-214-3p expression levels (% of control) were assessed. P values were calculated using One-way ANOVA. ##p < 0.01 vs. Control; **p< 0.01 vs. MI/R. (C) Sequence alignment predicted the potential binding site between miR-

214-3p and its target gene, Ghrl. The PCR results from the same batch of samples used for RNA sequencing showed that treatment with siMIAT-20mer-UU-3 reversed the upregulation of MIAT observed in the model group. It suggested that MIAT may act as a molecular sponge to regulate miR-214-3p, which is predicted to target the Ghrl gene based on the miRanda database. (D) Effects of siMIAT-20mer-UU-3 and miR-214-3p inhibitor on cell viability in H9c2 cells subjected to H/R conditions. Data are presented as means ± SDs from 3 independent experiments. (E) MIAT RNA expression was measured by quantitative PCR in H/R-injured H9c2 cells treated with: Control, H/R, miR-214-3p inhibitor, negative control inhibitor and siMIAT-20mer-UU-3. Data are presented as means ± SDs from 4 independent experiments. P values were calculated using one-way ANOVA. $*p < 0.05$, $**p < 0.01$, $***p < 0.001$, $****p < 0.0001$.

Table S1. Information of twenty optimized siRNAs.

siRNA Name	Passenger strand (5'-3')	Length (mer)	Guide strand (5'-3')	Length (mer)
siMIAT-19mer-1	UUUCAUGCCUCACCUCCAG	19	CUGGAGGUGAGGCAUGAAA	19
siMIAT-19mer-2	UUCAUGCCUCACCUCCAGU		ACUGGAGGUGAGGCAUGAA	
siMIAT-19mer-3	UCAUGCCUCACCUCCAGUA		UACUGGAGGUGAGGCAUGA	
siMIAT-19mer-4	CAUGCCUCACCUCCAGUAG		CUACUGGAGGUGAGGCAUG	
siMIAT-19mer-5	AUGCCUCACCUCCAGUAGG		CCUACUGGAGGUGAGGCAU	
siMIAT-19mer-UU-1	UUUCAUGCCUCACCUCCAGUU	21	CUGGAGGUGAGGCAUGAAAUU	21
siMIAT-19mer-UU-2	UUCAUGCCUCACCUCCAGUUU		ACUGGAGGUGAGGCAUGAAUU	
siMIAT-19mer-UU-3	UCAUGCCUCACCUCCAGUAUU		UACUGGAGGUGAGGCAUGAUU	
siMIAT-19mer-UU-4	CAUGCCUCACCUCCAGUAGUU		CUACUGGAGGUGAGGCAUGUU	
siMIAT-19mer-UU-5	AUGCCUCACCUCCAGUAGGUU		CCUACUGGAGGUGAGGCAUUU	
siMIAT-20mer-1	AUUUCAUGCCUCACCUCCAG	20	CUGGAGGUGAGGCAUGAAAU	20
siMIAT-20mer-2	UUUCAUGCCUCACCUCCAGU		ACUGGAGGUGAGGCAUGAAA	
siMIAT-20mer-3	UUCAUGCCUCACCUCCAGUA		UACUGGAGGUGAGGCAUGAA	
siMIAT-20mer-4	UCAUGCCUCACCUCCAGUAG		CUACUGGAGGUGAGGCAUGA	
siMIAT-20mer-5	CAUGCCUCACCUCCAGUAGG		CCUACUGGAGGUGAGGCAUG	
siMIAT-20mer-UU-1	AUUUCAUGCCUCACCUCCAGUU	22	CUGGAGGUGAGGCAUGAAAUUU	22
siMIAT-20mer-UU-2	UUUCAUGCCUCACCUCCAGUUU		ACUGGAGGUGAGGCAUGAAAUU	
siMIAT-20mer-UU-3	UUCAUGCCUCACCUCCAGUAUU		UACUGGAGGUGAGGCAUGAAUU	
siMIAT-20mer-UU-4	UCAUGCCUCACCUCCAGUAGUU		CUACUGGAGGUGAGGCAUGAUU	
siMIAT-20mer-UU-5	CAUGCCUCACCUCCAGUAGGUU		CCUACUGGAGGUGAGGCAUGUU	

Table S2. Primers for quantitative real-time PCR.

Gene name		Sequence of oligonucleotides
H-GAPDH	F	GTCTCCTCTGACTTCAACAGCG
	R	ACCACCCTGTTGCTGTAGCCAA
H-MIAT	F	GGACGTTCACAACCACACTG
	R	TCCCACTTTGGCATTCTAGG
R-GAPDH	F	TGCCCAGAACATCATCCCT
	R	GGTCCTCAGTGTAGCCCAAG
R-MIAT	F	ACCAGCAACGGAGTAGTGTG
	R	CACAGCCCCGGAATGAAGAGT
R-U6	RT	AACGCTTCACGAATTTGCGT
	F	CTCGCTTCGGCAGCACAT
	R	AACGCTTCACGAATTTGCGT
R-miR-214-3p	RT	GTCGTATCCAGTGCAGGGTCCGAGGTATTCGCACTGGATACGACCTGCCT
	F	GCGGCGGACAGCAGGCACAGAC
	R	ATCCAGTGCAGGGTCCGAGG

Abbreviations: RT = reverse transcription; F = forward; R = reverse.
