# Supplementary material for: Optimization of siRNA therapeutics targeting MIAT for cardioprotection in myocardial ischemia/reperfusion injury
Source: Mol Ther Nucleic Acids. 2025 Oct 18;36(4):102747. doi: 10.1016/j.omtn.2025.102747 (PMC12617760; doi:10.1016/j.omtn.2025.102747)
Supplement: Document S2. Article plus supplemental information [file mmc2.pdf]

Molecular Therapy
Nucleic Acids
Original Article

Optimization of siRNA therapeutics targeting MIAT for cardioprotection in myocardial ischemia/reperfusion injury

Xiao-Rong Ma,[1,3] Tong-Meng Yan,[1,2,3] Yu Pan,[1,2] and Zhi-Hong Jiang[1,2]

[1]School of Pharmacy, Faculty of Medicine & State Key Laboratory of Mechanism and Quality of Chinese Medicine, Macau Institute for Applied Research in Medicine and Health, Macau University of Science and Technology, Taipa, Macau 999078, China; [2]Guangdong-Macao Joint Laboratory for Innovative Drug Research on TCM and Natural-Derived Small RNAs, Macau University of Science and Technology, Taipa, Macau 999078, China

Myocardial ischemia/reperfusion (MI/R) injury is a leading cause of heart failure, and novel therapeutic strategies are urgently needed to improve patient outcomes. Long non-coding RNA (lncRNA) myocardial infarction-associated transcript (MIAT) has been implicated in exacerbating myocardial damage during ischemia, making it an attractive target for RNA interference. In this study, we designed and optimized small interfering RNAs (siRNAs) to silence MIAT expression and evaluated their cardioprotective effects *in vitro* and *in vivo*. Through bioinformatics analysis, we identified conserved target sites within MIAT sequences across humans, rats, and mice. We demonstrated that siRNA targeting MIAT, particularly siMIAT-20mer-UU-3, significantly improved cell viability in human and rat myocardial cells subjected to hypoxia/reoxygenation (H/R) injury, with dose-dependent effects. Furthermore, siMIAT treatment alleviated key pathological processes, including calcium overload, oxidative stress, and mitochondrial dysfunction. In a rat MI/R model, siMIAT-20mer-UU-3 significantly reduced infarct size by 49.26%, stabilized cardiac function, and improved histopathological features. Transcriptomic analysis revealed that siMIAT modulated the cAMP/protein kinase A (PKA) and NADPH/reactive oxygen species (ROS) pathways, highlighting its role in restoring myocardial homeostasis. These findings provide strong evidence for the therapeutic potential of MIAT-targeting siRNAs in treating MI/R injury and offer a comprehensive approach to the development of RNA-based therapeutics for cardiovascular diseases.

INTRODUCTION

Myocardial ischemia/reperfusion (MI/R) injury is a leading cause of acute cardiac events and heart failure worldwide.[1] Following ischemic events, when blood supply to the heart muscle is restored, paradoxically, it can lead to further myocardial injury due to the sudden reoxygenation and associated cellular stress. This injury is aggravated by factors like oxidative stress, inflammation, mitochondrial dysfunction, and calcium overload, all of which severely impair cardiac function and increase mortality risk.[2] According to the World Health Organization, ischemic heart disease is the top cause of death worldwide, responsible for 13% of all global deaths.[3] Despite advances in medical and surgical treatments, including drugs like metoprolol and procedures such as coronary artery bypass surgery, outcomes for many patients remain unsatisfactory.[4,5] These approaches primarily focus on improving blood flow to the coronary arteries, without paying attention to the ability of myocardial cells to resist injury and repair when the heart is damaged.[6–8] Therefore, there is a pressing need for novel and more effective therapies to reduce the damage caused by MI/R injury and improve patient outcomes.

Recent studies have identified long non-coding RNAs (lncRNAs) as key regulators of gene expression and cellular processes in various cardiovascular diseases, including MI/R injury.[9,10] LncRNAs have been shown to participate in the regulation of inflammation, apoptosis, fibrosis, and oxidative stress, all of which are critical in the pathophysiology of myocardial injury.[11,12] Among these, the myocardial infarction-associated transcript (MIAT) has emerged as a significant contributor to cardiac damage. It is upregulated during ischemic conditions and worsens myocardial injury.[13] Professor Ishii was the first to link high MIAT expression with myocardial injury.[14] Dr. Chen demonstrated *in vitro* that silencing MIAT with shRNA can alleviate injury.[15] Qu et al. confirmed through animal knockout studies that reducing MIAT effectively treats myocardial cardiac fibrosis.[16] These findings strongly support the potential of MIAT as a therapeutic target, making it an attractive candidate for developing effective anti-myocardial ischemia drugs.

Targeting MIAT using RNA interference (RNAi) technology, specifically with small interfering RNAs (siRNAs), represents a

Received 22 February 2025; accepted 15 October 2025;
https://doi.org/10.1016/j.omtn.2025.102747.

[3]These authors contributed equally

Correspondence: Zhi-Hong Jiang, School of Pharmacy, Faculty of Medicine & State Key Laboratory of Mechanism and Quality of Chinese Medicine, Macau Institute for Applied Research in Medicine and Health, Macau University of Science and Technology, Taipa, Macau 999078, China.
E-mail: zhjiang@must.edu.mo

© 2025 The Author(s).
Published by Elsevier Inc. on behalf of The American Society of Gene and Cell Therapy.
This is an open access article under the CC BY license (http://creativecommons.org/licenses/by/4.0/).

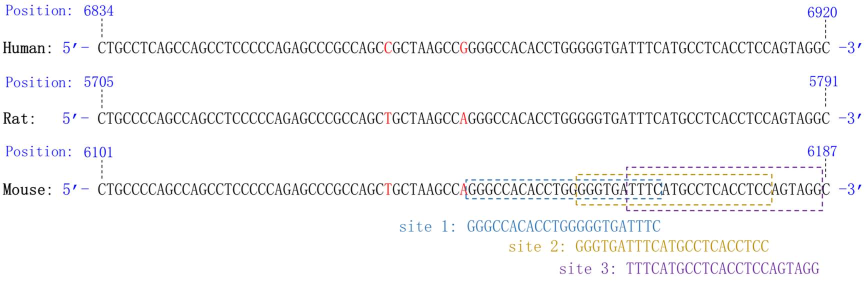

Figure 1. siRNA design sites
Homology regions of lncRNA MIAT sequences across three species and the selected target sites for siRNA design.

promising therapeutic strategy. RNAi enables precise gene silencing, offering high specificity and efficacy in modulating gene expression.[17] Since the discovery of RNAi in *Caenorhabditis elegans*, siRNA drugs have progressed from the lab to clinical applications.[18,19] Notably, Food and Drug Administration-approved siRNA therapeutics, such as Onpattro and Givlaari, have shown success in treating rare hereditary diseases, and several others are undergoing clinical evaluation.[20] While siRNA therapies have demonstrated promise in variety of diseases, such as cancer and viral infections, their application in cardiovascular diseases—particularly for targeting lncRNAs like MIAT—remains limited.[21] To date, although a few studies have investigated siRNAs targeting MIAT, no team has yet attempted to develop siRNA-based drugs specifically for MI/R injury.

In our previous study, we identified a tRNA-derived fragment, HC83, from ginseng, which exhibited significant activity against ischemia-reperfusion injury. We demonstrated that HC83 exerts its therapeutic effects by silencing the lncRNA MIAT.[22] This discovery introduced a new siRNA-based approach for treating MI/R injury and emphasized the potential of RNA-based drugs derived from traditional Chinese medicines (TCM), especially for diseases with unclear therapeutic targets. By tracing the effects of HC83 to MIAT, we confirmed MIAT as a promising target for RNAi in MI/R injury, laying the foundation for developing effective cardioprotective drugs. However, there are several key challenges in developing active RNAs like HC83 into drug candidates. First, species-specific differences in RNA sequences: genetic differences between species complicate the direct translation of findings from animal models to human therapies.[23] Even if effective RNA sequences are identified in animals, the genetic disparities can limit their effectiveness in humans, presenting a significant hurdle in RNA drug development. Second, incomplete sequence complementarity and off-target effects: TCM-derived RNA often does not match human sequences perfectly.[24] This mismatch reduces the silencing effect and increases the risk of off-target effects, making it more difficult to successfully translate these treatments into human therapies.

Building on our previous research, this study aims to address these challenges by optimizing HC83-derived siRNA to target MIAT. We focus on enhancing the efficacy and specificity of siRNA sequences to ensure effective silencing in both human and preclinical rodent models. Through this study, we aim to bridge the gap between discovering active RNA sequences from TCM and optimizing them into siRNA drugs suitable for human use. Additionally, we seek to establish a comprehensive framework for developing siRNA-based drugs from TCM, integrating traditional medicine with modern RNA therapeutics.

RESULTS

siRNA targeting MIAT provides significant protection to human cardiac cells

To investigate the protective effects of siRNA targeting lncRNA MIAT in preclinical models, we designed siRNAs based on conserved MIAT sequences shared among humans, rats, and mice. Bioinformatics analysis identified homologous regions in the MIAT sequences of these species: positions 6,834–6,920 in humans, 5,705–5,791 in rats, and 6,101–6,187 in mice (Figure 1). The left side of these regions had higher G/C content, while the right side (positions 6,873–6,920 in humans) was selected for siRNA construction to ensure target specificity. Three distinct target sites within this region were chosen for siRNA design, and an initial siRNA was designed for each site to assess their effectiveness (Table 1).

The protective effects of the three siRNAs were evaluated on the CCC-HEH-2 model. Confocal microscopy observations (Figure S1) showed that naked siRNAs effectively penetrated to hypoxia/reoxygenation (H/R)-injured cells without transfection reagents. Cells were treated with 300 nM siRNA before reoxygenation, and cell viability was assessed to measure the protective effects. As shown in Figure 2A, siMIAT-site3 significantly improved cell viability by 9.92% compared to the H/R group ($p < 0.001$), indicating robust protection against H/R injury. In comparison, siMIAT-site1 and siMIAT-site2 showed protective effects of 7.61% and 5.82%, respectively. Further analysis revealed a dose-dependent increase in cell viability with siMIAT-site3 treatment (Figure 2B). At 600 nM, siMIAT-site3 achieved a maximum protective effect of 14.46%, improving cell viability from 45.07% in the H/R group to 59.53%. These results demonstrate that siMIAT targeting site 3 provides significant protection to human myocardial cells under H/R conditions, highlighting its therapeutic potential for ischemia/reperfusion injury.

Optimization of siMIAT sequences enhances cardioprotection

Building on the protective effects of siMIAT-site3 at target site 3, we systematically optimized its sequence to further enhance its cardioprotective activity. Modifications included changes in sequence length, shifts in the target site, and adjustments to terminal overhangs, resulting in 20 additional siMIAT variants (Table S1).

Table 1. Three siRNA sequences were designed

siRNA name	Passenger strand (5′-3′)	Guide strand (5′-3′)
siMIAT – site1	GCCACACCUGGGGGUGAUUUU	AAUCACCCCCAGGUGUGGCUU
siMIAT – site2	GUGAUUUCAUGCCUCACCUUU	AGGUGAGGCAUGAAAUCACUU
siMIAT – site3	UCAUGCCUCACCUCCAGUAUU	UACUGGAGGUGAGGCAUGAUU

A comprehensive screening of these variants was conducted on the CCC-HEH-2 model at 300 nM. The results showed that all variants exhibited activity (Figure 2C), as they effectively targeted the MIAT, which has been previously identified as protective when downregulated. Our analysis also identified two key patterns that significantly influenced the activity of the siMIATs:

(1) Effect of 5′ end base on activity: To assess the impact of the 5′ end base on siRNA activity, we designed several 19-mer siRNAs targeting site 3. These siRNAs covered the entire region with different 5′ end bases by sliding along the template sequence. Results in Figure 3A showed that siRNAs with an A/U base at the 5′ end consistently exhibited higher activity than those with a G/C base. Specifically, the protective efficacy increased from 5.17% (C/G base) to 8.39% for A/U siRNAs (Figure 3A). This trend was confirmed with 20-mer siRNAs, where A/U siRNAs showed a further increase in efficacy, with protective efficacy rising from 5.16% to 9.10% (Figure 3A). Interestingly, the 3′ terminal base did not significantly affect activity. These findings suggest that the 5′ end base, particularly A/U, is key to enhancing siRNA efficacy.
(2) Effect of 3′ end overhang on activity: To assess the impact of the 3′ overhang on siRNA activity, we designed several 19-mer and 20-mer siRNAs targeting site 3 with a -UU overhang at the 3′ end. The results showed that adding the -UU overhang generally improved siRNA activity, with an average increase of 15.31% in activity and some sequences showing up to a 60% increase (Figure 3B). These findings suggest that the 3′ -UU overhang enhances siRNA activity and is useful for optimizing the cytoprotective effects of siMIAT.

Among all variants, siMIAT-20mer-UU-3 and siMIAT-20mer-3 showed the strongest protective effects in human myocardial cells (CCC-HEH-2). Both siRNAs demonstrated dose-dependent improvements in cell viability after H/R treatment. At 600 nM, siMIAT-20mer-UU-3 reached a maximum protective effect of 16.01%, increasing viability from 44.20% to 60.21% (Figure 4A). Similarly, siMIAT-20mer-3 achieved a 17.10% increase, raising cell viability from 43.55% to 60.65% (Figure 4B).

The protective effects of siMIAT-19mer-UU-3, siMIAT-20mer-3, and siMIAT-20mer-UU-3 were confirmed in rat cardiomyocytes (H9c2) subjected to H/R treatment. As in CCC-HEH-2 cells, all three siRNAs significantly improved H9c2 cell survival. Specifically, siMIAT-19mer-UU-3, siMIAT-20mer-3, and siMIAT-20mer-UU-3 increased cell viability to 62.52%, 60.68%, and 63.54%, respectively, compared to 51.05% in the H/R group ($p < 0.0001$) (Figure 4C). siMIAT-19mer-UU-3 and siMIAT-20mer-UU-3 showed more pronounced protective effects, with dose-dependent improvements in cell viability. At 600 nM, siMIAT-20mer-UU-3 improved it by 16.23% (from 46.39% to 62.62%), while siMIAT-19mer-UU-3 increased viability by 15.30% (from 47.45% to 62.75%) (Figures 4D and 4E). To exclude off-target effects and confirm species-specific targeting, we performed cross-species validation, demonstrating that siRNAs designed for human MIAT effectively silenced MIAT in CCC-HEH-2 cells, while those designed for rat MIAT were effective in H9c2 cells (Figure S2).

siMIAT restores MIAT expression to normal physiological levels in H/R-injured cardiomyocytes

To confirm that the protective effects of siMIAT are mediated through MIAT regulation, we measured MIAT levels in H/R-injured CCC-HEH-2 and H9c2 cells using qPCR. As shown in Figures 4F and 4G, MIAT expression was significantly upregulated in both cell types after H/R injury. In H/R-injured CCC-HEH-2 cells, MIAT increased by 108.51%–159.31% above control levels ($p < 0.0001$), but treatment with siMIAT-20mer-UU-3 and siMIAT-20mer-3 reduced MIAT expression to 65.80% and 21.52% above control levels, respectively ($p < 0.0001$). These results indicate that siMIAT-20mer-3 had a greater knockdown effect. In H9c2 cells, MIAT expression increased by 195.16%–223.18% ($p < 0.0001$) after H/R injury, but siMIAT-19mer-UU-3 and siMIAT-20mer-UU-3 reduced MIAT levels to 51.02% and 56.94% above control levels, respectively ($p < 0.0001$).

Collectively, these results confirm that the protective effects of siMIATs against H/R injury are directly linked to their ability to downregulate MIAT expression. Based on these findings, siMIAT-20mer-3 was selected for further mechanistic studies in CCC-HEH-2 cells, while siMIAT-20mer-UU-3 was chosen for *in vivo* experiments in a rat MI/R model. These data highlight the crucial role of MIAT silencing in reducing H/R-induced myocardial injury.

siMIAT alleviates pathological molecular processes in H/R-injured cardiomyocytes

To investigate whether siMIAT treatment reduces the pathological effects of MIAT upregulation, we evaluated key markers of myocardial injury in CCC-HEH-2 cells after H/R treatment. siMIAT-20mer-3 was chosen for these studies due to its superior cytoprotective effects.

(1) Intracellular calcium overload: H/R exposure increased intracellular calcium (Ca^{2+}) levels by 67.1% ($p < 0.01$) compared to the control group. Treatment with 300 nM siMIAT reduced

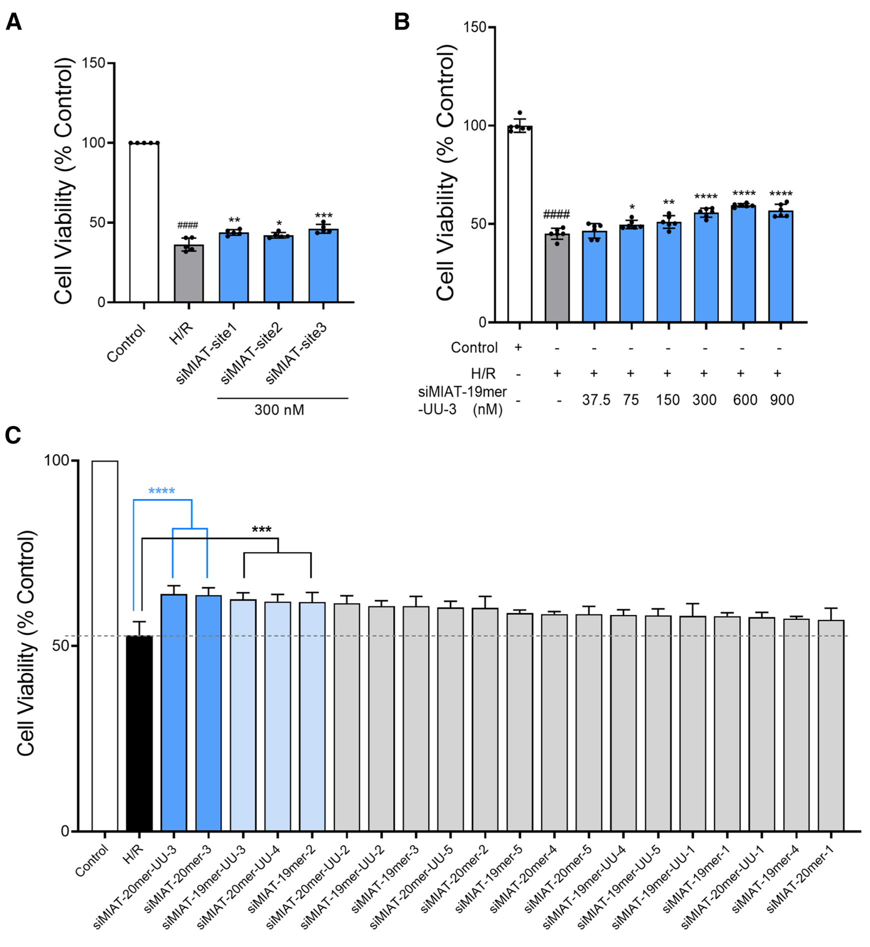

Figure 2. siMIAT treatment increases CCC-HEH-2 cell viability

(A) Activity of siMIAT-site1, siMIAT-site2, and siMIAT-site3 at 300 nM on CCC-HEH-2 cells. Data are presented as means ± SDs from 5 independent experiments. *p* values were calculated using one-way ANOVA. ####$p < 0.0001$ vs. control; *$p < 0.05$, **$p < 0.01$, ***$p < 0.001$ vs. H/R. (B) Dose-dependent effects of siMIAT-site3 ($n = 6$ per group). ####$p < 0.0001$ vs. control; *$p < 0.05$, **$p < 0.01$, ****$p < 0.0001$ vs. H/R. (C) Screening of 20 siRNAs on H/R-injured CCC-HEH-2 cells. Data are presented as means ± SDs from 3 independent experiments. ***$p < 0.001$, ****$p < 0.0001$ vs. H/R.

this increase to 10.1% above the control ($p < 0.01$ vs. H/R), suggesting that it prevents calcium dysregulation caused by H/R (Figures 5A and 5B).

(2) Reactive oxygen species (ROS) generation: ROS levels increased by 56.3% ($p < 0.01$) after H/R exposure. siMIAT at 300 nM suppressed this increase, with ROS rising only 3.7% compared to the control ($p < 0.01$ vs. H/R), indicating significant inhibition of ROS overproduction (Figures 5C and 5D).

(3) Mitochondrial membrane potential ($\Delta\Psi$m): H/R treatment caused a 26.4% decrease in $\Delta\Psi$m ($p < 0.001$), evidenced by a shift from red to green fluorescence, indicating mitochondrial dysfunction. siMIAT-20mer-3 treatment reduced this loss to 10.7% ($p < 0.01$ vs. H/R), mitigating mitochondrial damage (Figures 5E and 5F).

These results collectively show that siMIAT alleviates calcium overload, oxidative stress, and mitochondrial dysfunction in H/R-injured cardiomyocytes. By silencing MIAT, siMIAT restores cellular homeostasis, highlighting its potential in treating MI/R injury.

siMIAT alleviates MI/R-induced myocardial infarction *in vivo*

To evaluate the cardioprotective effects of siMIAT *in vivo*, siMIAT-20mer-UU-3, the most effective siRNA in H9c2 cells, was tested in a rat MI/R model. siMIAT-20mer-UU-3 was encapsulated in a peptide nanoparticle system and administered via tail vein injection. As shown in Figure 6A, siMIAT-20mer-UU-3 treatment significantly reduced the infarcted area. Quantitative analysis showed that the infarct area decreased from 32.40% ± 7.56% in the MI/R group to 16.44% ± 4.50% in the siMIAT-20mer-UU-3 treatment group (28 nmol/kg), corresponding to a 49.26% reduction ($p < 0.001$, Figure 6B).

Electrocardiogram (ECG) analysis further supported the cardioprotective effects of siMIAT-20mer-UU-3. Before surgery, ECG traces from all groups were normal. After coronary artery ligation, both the MI/R and siMIAT-20mer-UU-3 groups showed ST-segment elevation and abnormal QRS waveforms, indicating myocardial infarction. However, after siMIAT-20mer-UU-3 treatment, ST-segment elevation was reduced, and QRS morphology improved, suggesting partial recovery of cardiac function. In contrast, the MI/R group showed persistent abnormalities (Figures 6C, 6D, and S3). It is important to note that the observed ∼50% reduction in ST-segment elevation in the MI/R control group during reperfusion is consistent with the expected outcomes in typical MI/R models. This confirms that adequate reperfusion was achieved in our study.

Histopathological analysis confirmed these effects. Heart tissue from the MI/R group showed significant damage, including disrupted fibers, edema, and condensed nuclei. In contrast, tissue from siMIAT-20mer-UU-3-treated rats showed well-aligned fibers, reduced edema, and improved nuclear morphology (Figure 6E). Sham tissue showed normal cardiac structure.

Echocardiographic measurements (Figures 6F and 6G) demonstrated that siMIAT-20mer-UU-3 treatment partially restored

A

Human MIAT: 5'- CGCTAAGCCGGGGCCACACCTGGGGGTGATTTCATGCCTCACCTCCAGTAGGC - 3'

siMIAT-19mer-1	3'-	AAAGUACGGAGUGGAGGUC	5.14%
siMIAT-19mer-2	3'-	AAGUACGGAGUGGAGGUCA	8.96%
siMIAT-19mer-3	3'-	AGUACGGAGUGGAGGUCAU	7.82%
siMIAT-19mer-4	3'-	GUACGGAGUGGAGGUCAUC	4.43%
siMIAT-19mer-5	3'-	UACGGAGUGGAGGUCAUCC	5.94%

siMIAT-20mer-1	3'-	UAAAGUACGGAGUGGAGGUC	4.17%
siMIAT-20mer-2	3'-	AAAGUACGGAGUGGAGGUCA	7.36%
siMIAT-20mer-3	3'-	AAGUACGGAGUGGAGGUCAU	10.84%
siMIAT-20mer-4	3'-	AGUACGGAGUGGAGGUCAUC	5.69%
siMIAT-20mer-5	3'-	GUACGGAGUGGAGGUCAUCC	5.63%

B

siMIAT-19mer-1	3'-	AAAGUACGGAGUGGAGGUC	5.14%
siMIAT-19mer-UU-1	3'-	UUAAAGUACGGAGUGGAGGUC	5.19%
siMIAT-19mer-2	3'-	AAGUACGGAGUGGAGGUCA	8.96%
siMIAT-19mer-UU-2	3'-	UUAAGUACGGAGUGGAGGUCA	7.84%
siMIAT-19mer-3	3'-	AGUACGGAGUGGAGGUCAU	7.82%
siMIAT-19mer-UU-3	3'-	UUAGUACGGAGUGGAGGUCAU	9.65%
siMIAT-19mer-4	3'-	GUACGGAGUGGAGGUCAUC	4.43%
siMIAT-19mer-UU-4	3'-	UUGUACGGAGUGGAGGUCAUC	5.46%
siMIAT-19mer-5	3'-	UACGGAGUGGAGGUCAUCC	5.94%
siMIAT-19mer-UU-5	3'-	UUUACGGAGUGGAGGUCAUCC	5.31%

siMIAT-20mer-1	3'-	UAAAGUACGGAGUGGAGGUC	4.17%
siMIAT-20mer-UU-1	3'-	UUUAAAGUACGGAGUGGAGGUC	4.85%
siMIAT-20mer-2	3'-	AAAGUACGGAGUGGAGGUCA	7.36%
siMIAT-20mer-UU-2	3'-	UUAAAGUACGGAGUGGAGGUCA	8.63%
siMIAT-20mer-3	3'-	AAGUACGGAGUGGAGGUCAU	10.84%
siMIAT-20mer-UU-3	3'-	UUAAGUACGGAGUGGAGGUCAU	11.09%
siMIAT-20mer-4	3'-	AGUACGGAGUGGAGGUCAUC	5.69%
siMIAT-20mer-UU-4	3'-	UUAGUACGGAGUGGAGGUCAUC	9.10%
siMIAT-20mer-5	3'-	GUACGGAGUGGAGGUCAUCC	5.63%
siMIAT-20mer-UU-5	3'-	UUGUACGGAGUGGAGGUCAUCC	7.47%

Figure 3. Optimization of siMIAT sequences enhances cardioprotection

(A) Effect of 5′ end base on activity. (B) Effect of 3′ end overhang on activity.

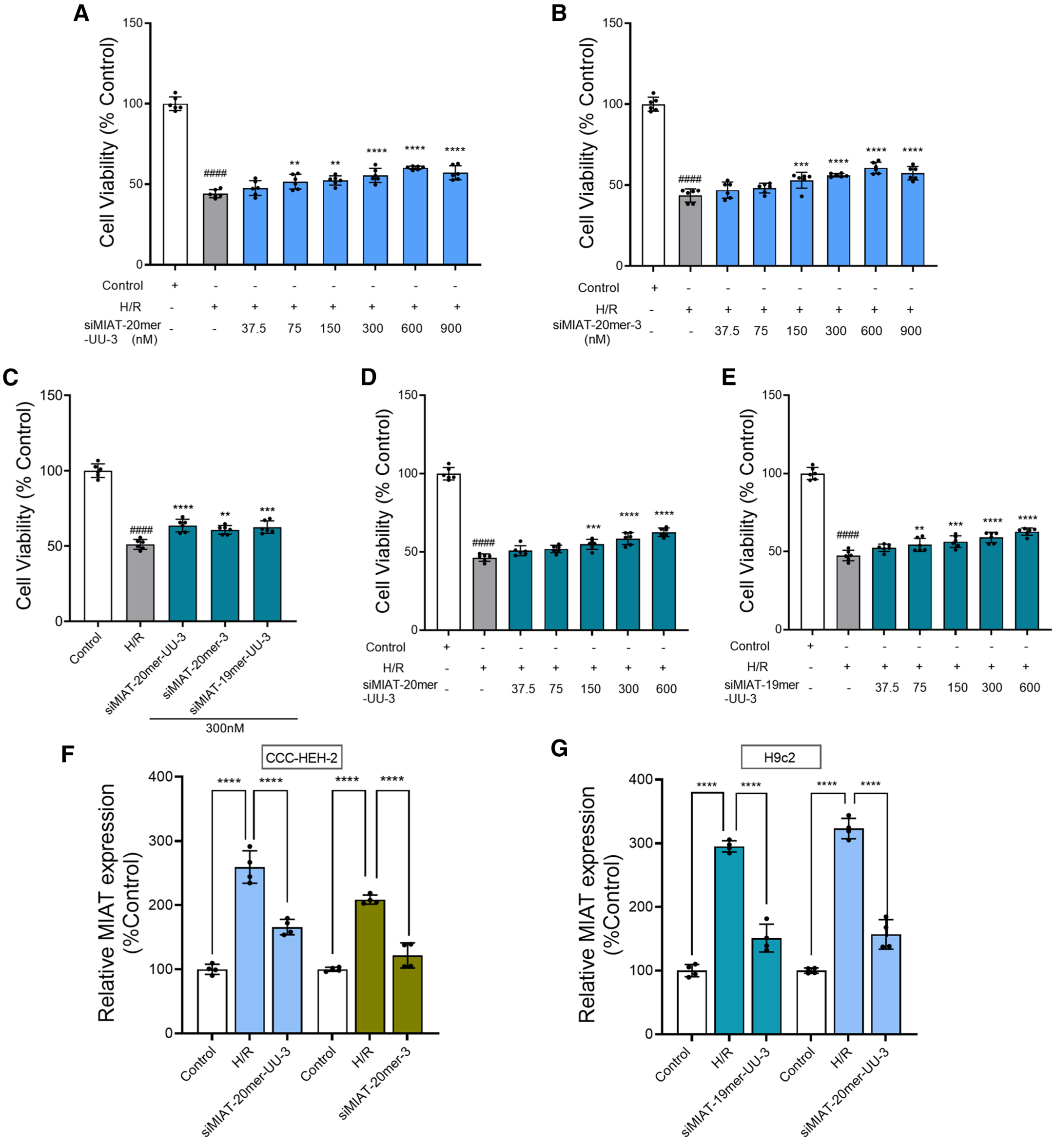

Figure 4. siMIAT enhances cell viability and reverses MIAT upregulation in H/R-injured cardiac cell lines

(A) Dose-dependent effects of siMIAT-20mer-UU-3 and (B) siMIAT-20mer-3 on CCC-HEH-2 cells (n = 6 per group). p values were calculated using one-way ANOVA. ####$p < 0.0001$ vs. control; **$p < 0.01$, ***$p < 0.001$, ****$p < 0.0001$ vs. H/R. (C) Activity of siMIAT-20mer-UU-3, siMIAT-20mer-3, and siMIAT-19mer-UU-3 on H9c2 cells at 300 nM (n = 6 per group). (D and E) Dose-dependent effects of siMIAT-20mer-UU-3 and siMIAT-19mer-UU-3 on H9c2 cells (n = 6 per group). ####$p < 0.0001$ vs. control; **$p < 0.01$, ***$p < 0.001$, ****$p < 0.0001$ vs. H/R. (F) MIAT RNA expression was measured by quantitative PCR in H/R-injured CCC-HEH-2 cells treated with 300 nM of siMIAT-20mer-UU-3 and siMIAT-20mer-3. (G) MIAT RNA expression was measured by quantitative PCR in H/R-injured H9c2 cells treated with 300 nM of siMIAT-19mer-UU-3 and siMIAT-20mer-UU-3. p values were calculated using one-way ANOVA. ****$p < 0.0001$ vs. H/R. Data are shown as the means ± SDs.

cardiac function in MI/R model rats. Compared with sham controls, the untreated MI/R group exhibited significantly impaired cardiac function, as indicated by marked reductions in ejection fraction (EF%, $p < 0.01$) and fractional shortening (FS%, $p < 0.01$). Administration of siMIAT-20mer-UU-3 facilitated substantial functional recovery, increasing EF% by 35.93% and FS% by 46.74% relative to untreated MI/R animals (both $p < 0.05$).

Serum biomarker analysis provided further confirmation of siMIAT-20mer-UU-3's cardioprotective effects (Figure 6H). Compared to sham controls, the MI/R group showed significant elevations in cardiac injury markers: cardiac troponin I (cTnI) levels increased by 28.08% ($p < 0.001$), creatine kinase-MB (CK-MB) by 64.46% ($p < 0.01$), and lactate dehydrogenase (LDH) by 58.27% ($p < 0.01$). Treatment with siMIAT-20mer-UU-3 effectively reduced these

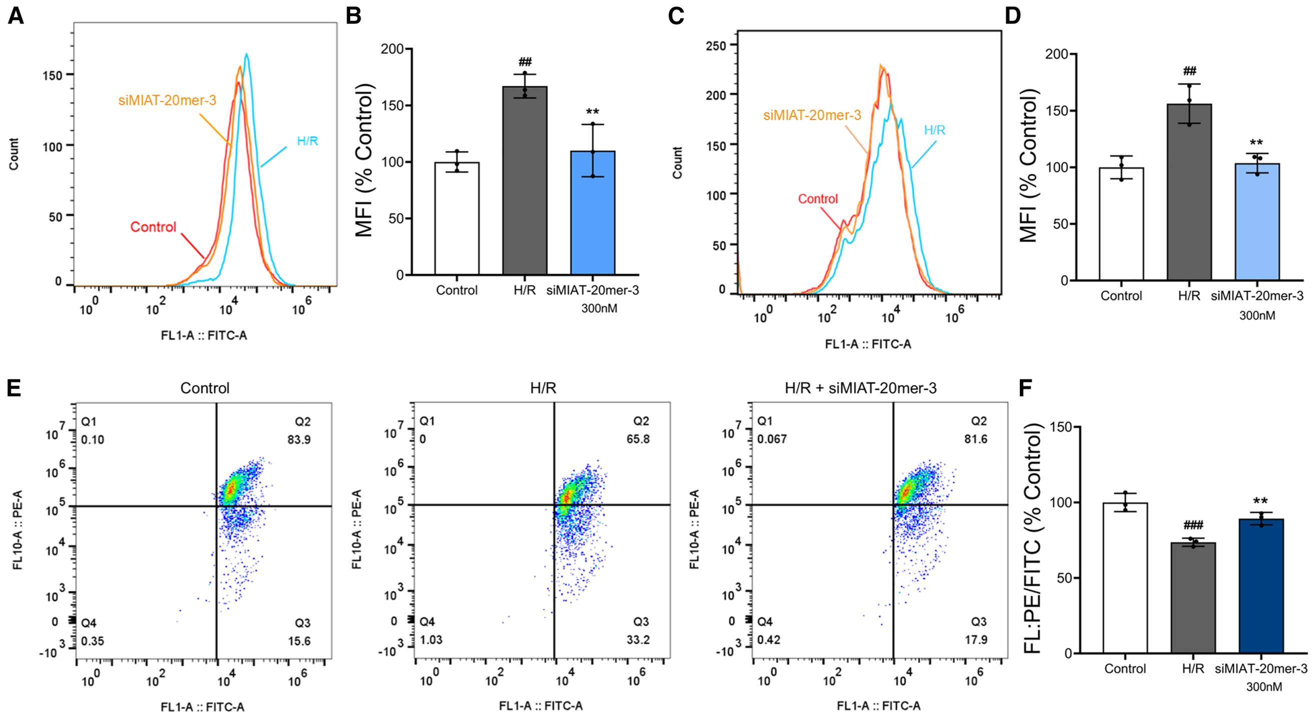

Figure 5. siMIAT-20mer-3 alleviates cell injury induced by H/R

(A) Intracellular Ca^{2+} levels were measured by flow cytometry. (B) Quantification of Ca^{2+} fluorescence intensity. (C) Intracellular ROS levels were measured by flow cytometry. (D) Quantification of ROS means fluorescence intensity. (E) Changes in mitochondrial membrane potential ($\Delta\Psi$m) induced by H/R were detected by flow cytometry. (F) Quantification of $\Delta\Psi$m expressed as the ratio of red to green fluorescence intensity. Data are presented as means ± SDs from 3 independent experiments. *p* values were calculated using one-way ANOVA. ##$p < 0.01$, ###$p < 0.001$ vs. control; **$p < 0.01$ vs. H/R.

pathological elevations, with cTnI showing a 16.23% decrease from MI/R levels ($p < 0.01$), resulting in concentrations only 7.30% higher than sham group values. Similar therapeutic efficacy was documented for both CK-MB (23.41% reduction from MI/R, $p < 0.05$; remaining 25.96% above sham) and LDH (26.37% reduction from MI/R, $p < 0.05$; 16.54% above sham levels).

These results demonstrate that siMIAT-20mer-UU-3 effectively reduces infarct size, stabilizes cardiac function, and mitigates histological damage, underscoring its potential as a therapeutic strategy for MI/R injury.

siMIAT restores MIAT expression levels in the MI/R-induced rat model

To assess the ability of siMIAT-20mer-UU-3 to silence MIAT expression *in vivo*, we measured MIAT levels in both infarcted and non-infarcted regions of rat hearts from the MI/R model. Given the documented regional heterogeneity in myocardial gene expression, we normalized MIAT levels using GAPDH specifically quantified from each corresponding zone. The infarcted regions were clearly distinguishable from the non-infarcted regions (Figure 6I), and MIAT expression was quantified in both areas. As illustrated in Figure 6J, MIAT expression was significantly higher in the MI/R group compared to the normal group. In the infarcted region, MIAT levels increased by 570.88% ($p < 0.0001$), and in the non-infarcted region, they rose by 168.15% ($p < 0.05$). These results indicate widespread MIAT upregulation after MI/R injury. Treatment with siMIAT-20mer-UU-3 reduced MIAT expression in a dose-dependent manner. In the infarcted region, the highest dose (28 nmol/kg) decreased MIAT expression from 570.88% to 189.75% above normal ($p < 0.001$), and in the non-infarcted region, it decreased from 168.15% to 59.53% above normal. These findings confirm that siMIAT-20mer-UU-3 effectively targets MIAT expression in both infarcted and surrounding tissues, with a stronger effect in the infarcted region. This suppression of MIAT is consistent with the observed cardioprotective effects, validating its therapeutic potential in MI/R injury.

Transcriptomic analysis reveals key pathways mediated by siMIAT in the MI/R-induced rat model

To investigate the molecular mechanisms of myocardial injury and the cardioprotective effects of siMIAT-20mer-UU-3, we performed RNA sequencing (RNA-seq) on heart tissues from the sham, MI/R, and siMIAT-20mer-UU-3 treatment groups (14 and 28 nmol/kg) ($n = 3$ per group). This analysis revealed significant gene expression changes associated with myocardial injury and repair. The MI/R group showed 3,147 upregulated and 1,990 downregulated genes compared to the sham group ($|\log2$ fold change$| \geq 1$, false discovery

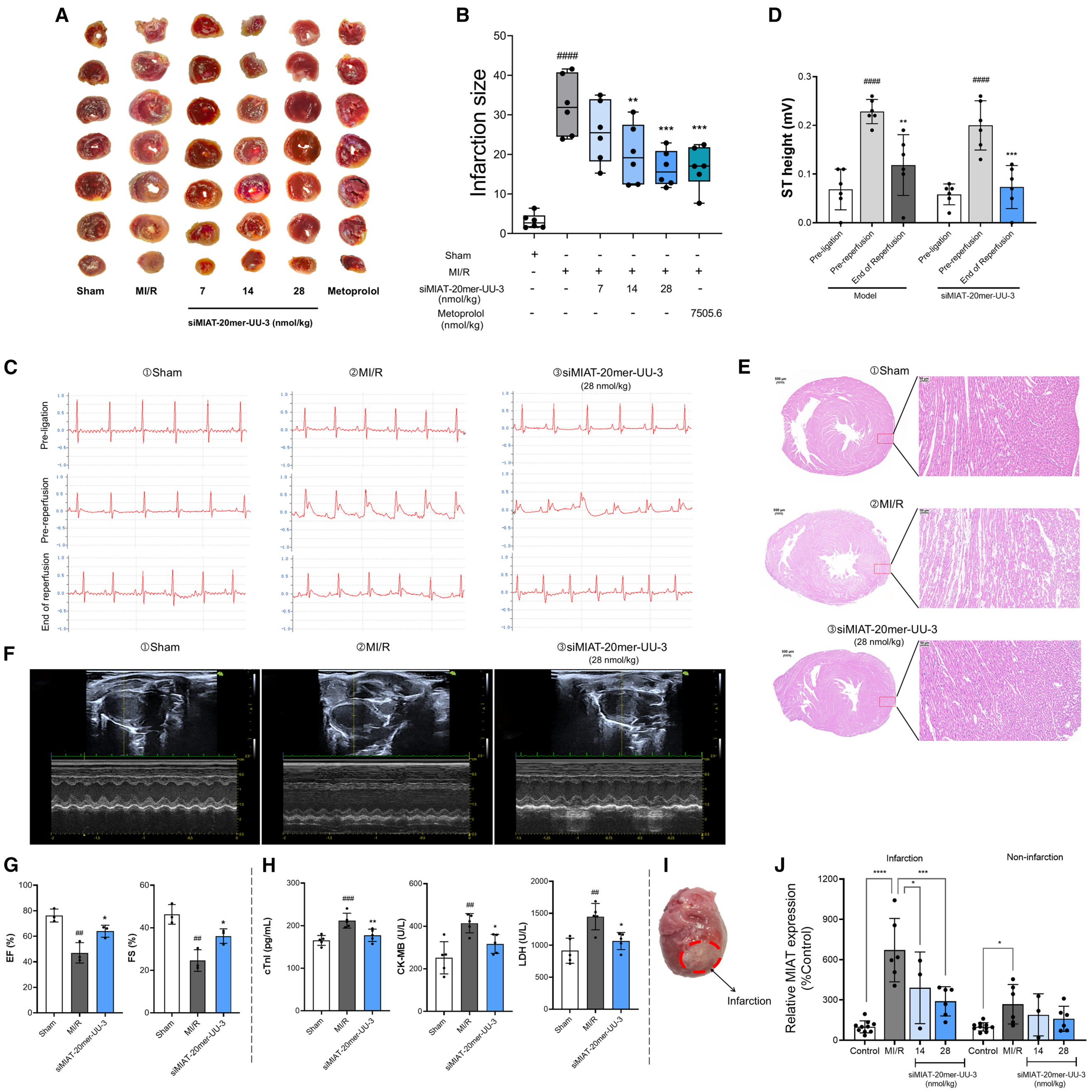

Figure 6. siMIAT-20mer-UU-3 protects the heart against MI/R injury *in vivo*

(A) Representative images of TTC-stained myocardial infarction tissues, with white areas indicating infarcted myocardium. (B) Quantification of infarction size. Data are presented as means ± SDs (n = 6). One-way ANOVA used for p value calculation. ####$p < 0.0001$ vs. sham, **$p < 0.01$, ***$p < 0.001$ vs. MI/R. (C) ECG comparisons across sham, MI/R, and siMIAT-20mer-UU-3 treatment groups (28 nmol/kg). (D) ST-segment elevation dynamics. Data are displayed as means ± SDs (n = 6). Statistical significance: ####$p < 0.0001$ vs. pre-ligation (within group); **$p < 0.01$, ***$p < 0.001$ vs. pre-reperfusion (within group). (E) Representative H&E-stained micrographs of heart tissue sections from sham, MI/R, and siMIAT-20mer-UU-3 treatment groups. (F) Representative M-mode echocardiography images from sham, MI/R, and siMIAT-20mer-UU-3 treatment groups following MI/R injury in rats. (G) Quantification of left ventricular ejection fraction (EF%) and fractional shortening (FS%) (n = 3). Data are expressed as means ± SDs. ##$p < 0.01$ vs. sham group, *$p < 0.05$ vs. MI/R group. (H) Serum levels of cardiac injury markers (cTnI, CK-MB, and LDH) post MI/R in experimental groups (n = 5). Data are shown as the means ± SDs. ##$p < 0.01$, ###$p < 0.001$ vs. sham; *$p < 0.05$, **$p < 0.01$ vs. MI/R. (I) Myocardial infarction in a rat heart post MI/R, with the infarcted region outlined in red. (J) MIAT RNA expression in infarct and non-infarct areas (n = 3–10). One-way ANOVA used for p value calculation. *$p < 0.05$, ***$p < 0.001$, ****$p < 0.0001$ vs. MI/R. Data are shown as the means ± SDs.

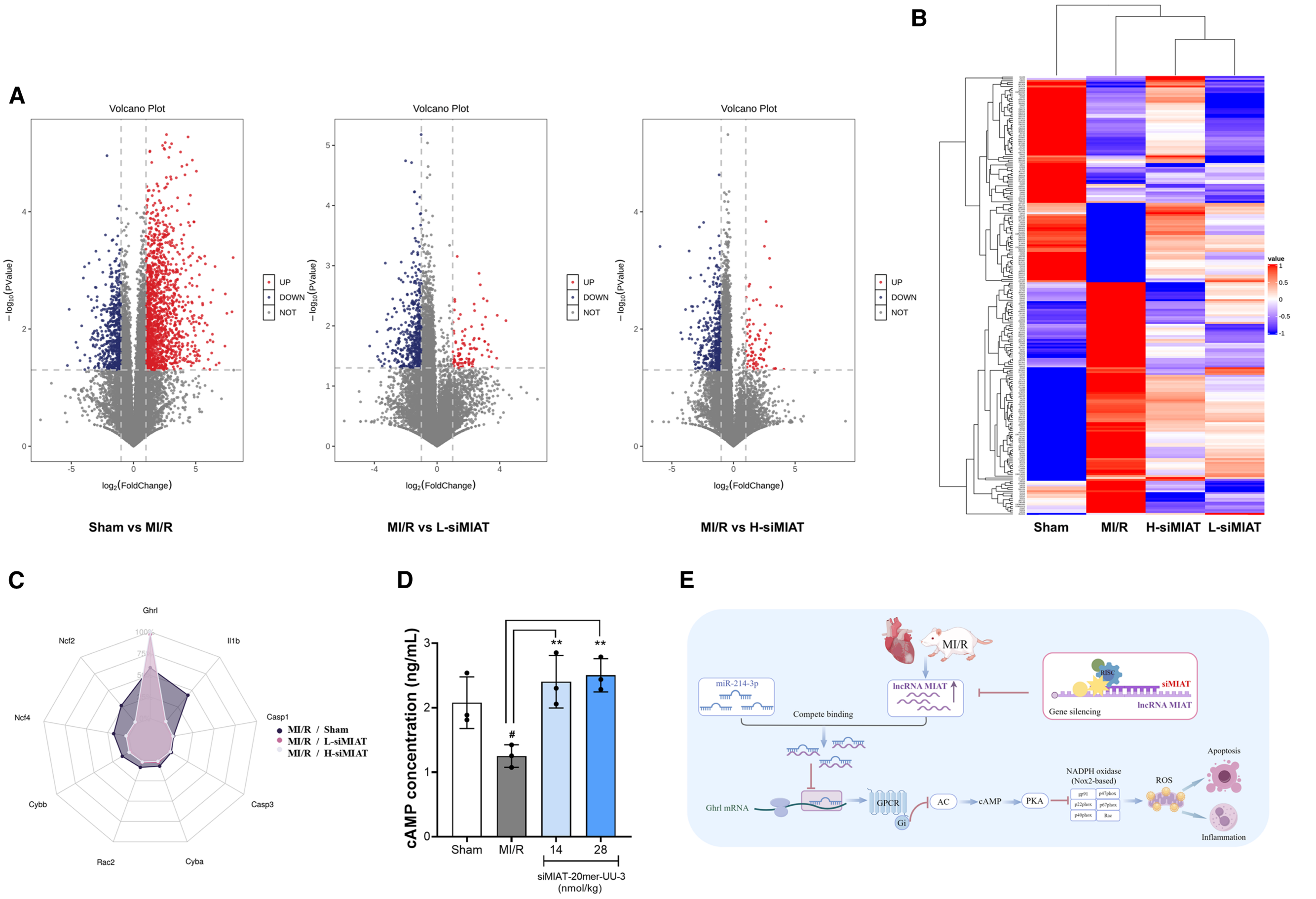

Figure 7. Analysis of mechanisms underlying myocardial injury and repair

(A) Volcano plots of differential gene expression: sham vs. MI/R (left), MI/R vs. 14 nmol/kg siMIAT-20mer-UU-3 (L-siMIAT, middle), and MI/R vs. 28 nmol/kg siMIAT-20mer-UU-3 (H-siMIAT, right). Red dots represent upregulated genes, blue dots indicate downregulated genes, and gray dots show genes with no significant change. (B) Heatmap of differentially expressed genes across the sham, MI/R, H-siMIAT, and L-siMIAT groups. The color gradient indicates gene expression levels from low (blue) to high (red). (C) Radar chart comparing the expression levels of selected genes involved in key cellular processes between MI/R and siMIAT groups. (D) Bar graph showing cAMP concentrations in different treatment groups. Data are shown as the means ± SDs. #$p < 0.05$ vs. sham, **$p < 0.01$ vs. MI/R. (E) Schematic diagram illustrating the potential molecular mechanisms by which siMIAT exerts its protective effects, modulating the cAMP/PKA and NADPH/ROS pathways, influencing inflammation and apoptosis.

rate [FDR] < 0.05) (Figure 7A). When compared to the MI/R group, the low-dose (14 nmol/kg) siMIAT-20mer-UU-3 group showed 199 upregulated and 1,878 downregulated genes (|log2 fold change| $\geq$ 1, FDR < 0.05). Similarly, compared to the MI/R group, the high-dose (28 nmol/kg) siMIAT-20mer-UU-3 group had 167 upregulated and 2,656 downregulated genes.

We identified 236 differentially expressed genes potentially linked to myocardial injury and repair (Figure 7B). Pathway analysis highlighted the involvement of the cAMP/protein kinase A (PKA) and NADPH/ROS pathways, both related to oxidative stress and myocardial protection (Figure 7C). In the cAMP/PKA pathway, the Ghrl gene, upregulated by 17.8-fold in the MI/R group, was normalized in the siMIAT-20mer-UU-3-treated groups. In the NADPH/ROS pathway, genes encoding NADPH oxidase components were upregulated in the MI/R group but downregulated with siMIAT-20mer-UU-3 treatment in a dose-dependent manner. Notably, Cybb (gp91phox/NOX2) decreased from 3.65-fold in the MI/R group to 1.47-fold in the high-dose siMIAT-20mer-UU-3 group, and other NADPH oxidase genes, such as Ncf2, Ncf4, and RAC2, showed similar reductions.

Apoptotic markers were also assessed, showing significant reductions after siMIAT-20mer-UU-3 treatment. Caspase-3, upregulated by 91.1% in the MI/R group, was reduced to 41.7% (low-dose) and 30.4% (high-dose) above normal levels. Caspase-1 also decreased significantly with siMIAT-20mer-UU-3 treatment. These findings suggest that siMIAT-20mer-UU-3 reduces apoptosis and cardiac remodeling in the MI/R model.

Overall, these RNA-seq results provide insights into the mechanisms underlying siMIAT-20mer-UU-3's cardioprotective effects. By modulating the cAMP/PKA and NADPH/ROS pathways and suppressing

apoptotic genes, siMIAT-20mer-UU-3 helps restore myocardial homeostasis and mitigate MI/R-induced damage.

siMIAT restores cAMP levels in the MI/R-induced rat model

To validate the cAMP/PKA pathway suggested by transcriptomic analysis, cAMP levels were measured in heart tissue samples. The results showed a significant reduction in cAMP levels in the MI/R group (1.25 ng/mL) compared to the sham group (2.08 ng/mL). Treatment with siMIAT-20mer-UU-3 restored cAMP levels in a dose-dependent manner. In the low-dose (14 nmol/kg) group, cAMP levels increased to 2.40 ng/mL, a 92% improvement over the MI/R group. The high-dose (28 nmol/kg) group showed further improvement, with cAMP levels rising to 2.51 ng/mL, a 100% increase ($p < 0.01$ vs. MI/R) (Figure 7D). These results support the proposed cAMP/PKA signaling pathway as a mechanism for the therapeutic effects of siMIAT-20mer-UU-3. As shown in Figure 7E, the molecular signaling cascade is influenced by siMIAT-20mer-UU-3, offering further insight into its cardioprotective actions. By restoring cAMP levels, siMIAT-20mer-UU-3 may enhance crucial signaling processes for cardiac repair and recovery after MI/R injury.

DISCUSSION

Here, we initially provide definitive evidence that silencing MIAT with siRNA significantly improves cell viability in both human and rat cardiomyocyte models subjected to H/R injury. We improved the efficacy of the siMIAT sequences from 5% to 17% (e.g., siMIAT-20mer-3 and siMIAT-20mer-UU-3, Figure 2), a notable enhancement considering the constraints of the H/R model, which typically allows only a 30% window to assess therapeutic efficacy.[25,26] This improvement marks these sequences as promising candidates for drug development. Specifically, the optimized siRNA sequence, siMIAT-20mer-UU-3, demonstrated even enhanced *in vivo* activity, laying a solid foundation for clinical translation. This siRNA effectively restored MIAT expression in the damaged region to physiological levels and reduced infarct size by 49.26%. Echocardiographic analysis revealed improvements in cardiac function, including a 35.93% increase in ejection fraction and a 46.74% enhancement in fractional shortening. Serum biomarkers further supported the therapeutic efficacy, with cTnI levels normalizing to within 7.30% of sham controls, accompanied by significant reductions in CK-MB and LDH. Consistent with these findings, the treatment also preserved histopathological architecture, restored physiological electrophysiological properties, and maintained overall cardiac function (Figure 6), further advancing the potential for myocardial ischemia therapeutics.

Through further optimization, we identified key design patterns that significantly impact siRNA efficacy. Specifically, G/C pairs at the 5′ end of the siRNA sequence reduced its cardioprotective activity, while a -UU overhang at the 3′ end greatly improved its therapeutic potential. These findings provide valuable insights into designing MIAT-targeting siRNA. Additionally, the consistent observation of these patterns strengthens the reliability and promise of the cardioprotective effects of siMIAT.

In our study, the cardioprotective effect of siMIAT-site3 peaked at 600 nM, with no further improvement at higher doses. This plateau likely reflects three factors: first, heterogeneous cell sensitivity in the H/R model, where some cells are irreversibly damaged and cannot respond to treatment; second, saturation of the RNA-induced silencing complex (RISC), which has limited capacity for siRNA loading and cannot increase activity once filled[18,19]; and third, limits in cellular uptake, as siRNA entry plateaus at high extracellular levels, leaving excess molecules outside the cells.[27,28] To overcome these barriers, improving delivery systems, enhancing RISC-loading efficiency, and targeting more responsive cell populations may extend the effective range and strengthen therapeutic outcomes.

A critical aspect of our study was the identification of optimal MIAT-targeting sites that are effective across mouse, rat, and human models. While animal models are commonly used in preclinical studies, the structural similarities of drug targets across species allow for extrapolation of findings, especially for small-molecule drugs, where targets (primarily proteins) are highly conserved.[29] However, siRNA drugs require precise matches between the siRNA and target RNA sequences to maximize efficacy and minimize off-target effects. This poses a challenge, as species-specific differences in RNA sequences can make animal findings unreliable for human therapy. Ensuring homology between animal models and human target regions is, therefore, crucial for accurate preclinical studies. The challenge in targeting MIAT arises from significant sequence variation across species. The human MIAT sequence spans 10,078 nucleotides, while the mouse and rat sequences are about 8,500 nucleotides long, with only a small, highly homologous region of 87 nucleotides, representing just 1% of the human MIAT sequence (Figure S4). Despite this, we identified three potential target sites, with site 3 emerging as the most promising for therapeutic efficacy. This finding is crucial for advancing siRNA-based drugs targeting MIAT, enabling preclinical evaluation of pharmacodynamics and toxicity despite species-specific differences.

Additionally, confirming homologous design regions between humans and rodents is essential for developing siRNA-based drugs derived from TCM. Even when effective RNA sequences from TCM are identified in animals, genetic discrepancies between species can render them ineffective in humans, creating significant barriers to RNA-based drug development. In our study, we addressed this challenge by optimizing the drug development process based on the MIAT target. Our strategy focuses on discovering bioactive RNA sequences from TCM, identifying relevant targets, and designing siRNA drugs applicable across species, particularly for both animal models and human applications. This approach aims to improve siRNA efficacy, minimize off-target effects, and overcome the species-specific challenges that have traditionally hindered RNA-based drug development from TCM.

In examining the molecular pathways involved in MIAT silencing, we found that silencing MIAT *in vivo* resulted in significant changes in Ghrl expression. Specifically, MIAT silencing reduced Ghrl expression, highlighting the “MIAT-Ghrl” axis as a key regulatory

pathway for therapeutic effects. Previous studies have shown that MIAT acts as a competing endogenous RNA, modulating miRNA expression, particularly miR-214-3p.[30] In our study, we confirmed a reciprocal regulatory relationship between MIAT and miR-214-3p, with siRNA treatment restoring MIAT expression and increasing miR-214-3p levels (Figure S5). Bioinformatics analysis predicted Ghrl as a target of miR-214-3p, suggesting that MIAT regulates Ghrl through miR-214-3p. The role of Ghrl in cardiac injury is underexplored, but its known function of inhibiting cAMP levels is critical. Specifically, Ghrl binds to G protein-coupled receptors, activating Gi proteins that suppress adenylate cyclase, thereby reducing cAMP production.[31,32] Our data show that Ghrl levels increase while cAMP levels decrease in the MI/R model, and after siMIAT-20mer-UU-3 treatment, Ghrl expression returned to normal and cAMP levels increased, indicating restored cellular function. These findings suggest that the "Ghrl-cAMP" axis plays a crucial role in myocardial injury and repair. It has been reported that reduced cAMP levels impair PKA activity, exacerbating oxidative stress and cardiac dysfunction.[33,34] Therefore, we propose the "siRNA-MIAT-miRNA-Ghrl-cAMP-PKA" pathway (Figure 7E), where upregulated MIAT reduces miR-214-3p, weakening its regulation of Ghrl, which leads to Ghrl upregulation. This in turn activates Gi proteins, suppresses cAMP, and impairs PKA activity, aggravating myocardial damage. Functional rescue experiments in H9c2 cells under H/R injury confirmed this mechanism. Co-administration of an miR-214-3p inhibitor significantly weakened the cardioprotective effects of siMIAT-20mer-UU-3 ($p < 0.01$) without altering MIAT expression (Figure S5). This establishes miR-214-3p as a key downstream effector in the pathway. Although these *in vitro* results are clear, further *in vivo* studies are needed to confirm the physiological importance of this regulatory axis in the whole heart. Considering the limited specificity of miR-214-3p inhibitors and their inability to achieve sufficiently sustained inhibition of miR-214-3p expression in animal models, generating miR-214-3p knockout mice may provide a more stable and physiologically relevant model to validate our proposed mechanism. Importantly, our findings provide preliminary evidence that targeting MIAT with siRNA restores balance in this pathway, reduces damage, and improves cardiac function. This offers new insights into MI/R injury and highlights potential therapeutic targets for activating the heart's own repair capacity.

Our study shows that siMIAT protects the heart mainly by targeting the lncRNA MIAT and enhancing the intrinsic repair mechanisms of heart cells, rather than by directly changing blood flow or hemodynamics. This mechanism is fundamentally different from that of conventional anti-ischemic drugs such as beta-blockers, nitrates, and calcium channel blockers. However, the actions of siMIAT could complement traditional drugs. While standard therapies reduce oxygen demand, widen coronary arteries, or prevent clotting,[35] siMIAT works at the genetic level to enhance repair and regeneration. This complementary effect suggests strong potential for synergistic therapies. For example, combining siMIAT with nitrates (e.g., nitroglycerin), which increase coronary blood flow and relieve ischemia,[36] could further enhance tolerance of heart cells to hypoxia-reoxygenation injury. This dual effect would optimize oxygen supply while also improving cell survival and repair. Similarly, combining siMIAT with ACE inhibitors (e.g., captopril), which lower ventricular afterload and reduce remodeling, could be particularly effective. ACE inhibitors lower angiotensin II levels and reduce fibrosis, while siMIAT may directly counter fibrosis and boost repair.[37,38] Together, they could prevent adverse remodeling after myocardial injury more effectively than either treatment alone. These findings suggest that siMIAT-20mer-UU-3 combined with traditional drugs could create a new therapeutic model: "blood flow optimization + cellular repair." Such an approach holds great promise for improving treatment outcomes in cardiovascular disease. However, the detailed molecular mechanisms of these combinations, as well as the best timing and dosing strategies, still need systematic study to maximize clinical benefits.

Furthermore, in this study, metoprolol was implemented as an active comparator or benchmark agent. Although beta-blockers are widely used as positive controls in basic research models of myocardial ischemia, their clinical relevance as an efficacy reference for acute ischemia-reperfusion injury remains controversial, as evidenced by discrepant conclusions from clinical trials such as METOCARD-CNIC and EARLY-BAMI.[39,40] Crucially, sensitivity analysis demonstrated that the core conclusion that siMIAT significantly reduces infarct size and improves cardiac function remains robust under scenarios that either include or exclude the metoprolol control data. The primary value of this study lies in establishing the inherent therapeutic efficacy of siMIAT itself. To enhance the translational relevance of future research, future studies will employ alternative comparators (e.g., diltiazem or amlodipine) or multiple reference agents.

Interestingly, we observed that siMIAT treatment significantly reduced MIAT expression throughout the heart. This effect was stronger in the damaged regions, while the undamaged regions showed only limited response. This matches our *in vitro* results, which showed that siRNA can enter injured heart cells without a delivery system but not healthy cells. These findings suggest that myocardial injury increases cell permeability, which in turn improves siRNA delivery to damaged areas. This difference in cell permeability may be largely driven by inflammation and oxidative stress. During the acute phase of I/R injury, immune cells such as neutrophils and macrophages release inflammatory cytokines (e.g., tumor necrosis factor alpha and interleukin-6) and adhesion molecules (e.g., ICAM-1 and VCAM-1).[41] These weaken the endothelial barrier, allowing macromolecules like siRNA to pass into the tissue. At the same time, oxidative stress generates ROS, which activate pathways such as mitogen-activated protein kinase and nuclear factor κB. These pathways damage endothelial cells, disrupt tight junctions, and further increase vascular permeability, making it easier for siRNA to enter the injured tissue.[42] These permeability changes may be closely linked to membrane proteins, especially Caveolin-1, which regulates endothelial permeability and signal transduction. In I/R injury, Caveolin-1 expression is altered, leading to overactivation of endothelial nitric oxide synthase.[43,44] This raises vascular permeability and enhances siRNA entry into damaged heart tissue.

On one hand, these results suggest that targeting key regulators of endothelial permeability, such as Caveolin-1, could further improve siRNA delivery to damaged areas and boost therapeutic efficacy. On the other hand, they highlight the need for efficient, heart-specific delivery systems to ensure that siRNA also reaches undamaged regions. Such systems could provide "preemptive protection" to high-risk areas and improve overall outcomes. Optimizing delivery systems in this way could offer broader cardiac protection during myocardial infarction, enhancing both short-term and long-term therapeutic effects. Therefore, systematic research into these mechanisms is essential. A better understanding of how pathological changes influence siRNA uptake will allow us to refine targeted delivery, maximize therapeutic benefit, minimize off-target effects, and significantly improve the precision and effectiveness of RNA-based therapies for myocardial injury.

In conclusion, our study demonstrates the potential of siRNA-based therapies targeting MIAT as a promising treatment for MI/R injury. Through the optimization of siRNA design and delivery, particularly with the siMIAT-20mer-UU-3 sequence, we have significantly enhanced therapeutic efficacy, safety, and specificity. Our findings emphasize the critical role of the "MIAT-Ghrl" axis in myocardial injury and repair, providing new insights into the molecular mechanisms underlying cardioprotection. We also addressed challenges related to species-specific differences, which have previously impeded the translation of animal model data to human therapies. This work offers a comprehensive framework for developing siRNA-based therapeutics derived from TCM, advancing both the understanding and clinical application of these therapies. By optimizing delivery systems and refining siRNA targeting, we believe that this approach holds the potential to significantly improve therapeutic outcomes in myocardial infarction and other cardiovascular diseases, offering a viable pathway for clinical intervention.

MATERIALS AND METHODS

Chemicals and reagents

Trizol reagent was obtained from Thermo Fisher Scientific (USA). Oligonucleotides were custom-synthesized by Biosyntech Co., Ltd. (Suzhou, China). Dulbecco's modified Eagle's medium (DMEM), glucose-free DMEM, fetal bovine serum (FBS), penicillin, and streptomycin were sourced from Gibco (New Zealand). Dichlorofluorescein diacetate (CM-H2DCFDA; C6827), Fluo-4 AM calcium indicator (F14201), and the MitoProbe JC-1 Assay Kit (M34152) were purchased from Invitrogen (USA). ELISA kits for cTn-I (E-EL-R1253) and cAMP quantification (E-EL-R0056) were purchased from Elabscience (China). miR-214-3p inhibitor (miR20000885-1-5) was purchased from RiboBio (China). All other chemicals were used as received.

Design and synthesis of siRNAs

We designed 22 siRNAs to target MIAT sequences across mouse, rat, and human, based on data from the NCBI database. The human MIAT sequence is listed under accession number GenBank: NR_003491, while the rat and mouse sequences are GenBank: NR_111959 and NR_033657, respectively. The siRNAs were custom-synthesized by Biosyntech Co., Ltd. (Suzhou, China) using solid-phase chemical synthesis. Their sequences were verified by liquid chromatography-mass chromatography, and their purity was confirmed through NanoDrop spectrophotometry and polyacrylamide gel electrophoresis.

Cell culture and H/R treatments

The human embryonic myocardial cell line CCC-HEH-2 was obtained from the National Experimental Cell Resources Sharing Service Platform (Kunming, China), and the rat cardiomyocyte-derived cell line H9c2 was purchased from the ATCC. Both cell lines were cultured in DMEM supplemented with 10% FBS and 1% penicillin/streptomycin in a humidified incubator at 37°C with 5% CO_2.

For hypoxic treatment, CCC-HEH-2 cells were incubated in glucose-free DMEM in a hypoxic chamber (Whitley H35 Hypoxystation) with 94% N_2, 5% CO_2, and 1% O_2 for 10 h. H9c2 cells were similarly incubated in glucose-free DMEM with 94.9% N_2, 5% CO_2, and 0.1% O_2 for 12 h. Afterward, cells were reoxygenated by incubating in complete growth medium under normoxic conditions for 6 h. Drugs were added to the medium before reoxygenation and maintained throughout the reoxygenation period to ensure consistent exposure.

Cell viability assay

The cell viability assay was performed according to the manufacturer's instructions. CCC-HEH-2 and H9c2 cells were seeded into 96-well plates at 5,000 cells per well and cultured for 24 h before hypoxic treatment. After H/R treatment, 100 μL of MTT solution (0.5 mg/mL) was added to each well and incubated at 37°C for 4 h. Then, 150 μL DMSO was added to dissolve the formazan crystals. The optical density (OD) at 570 nm was measured using a SpectraMax Paradigm microplate reader, and these values were used to assess cell viability.

Quantitative real-time PCR analysis

Total RNA was extracted from myocardial cells or rat heart tissues using Trizol reagent. The RNA was reverse transcribed into complementary DNA with the RevertAid First Strand cDNA Synthesis Kit (Thermo Fisher Scientific). Quantitative real-time PCR was performed on a ViiA 7 system (Life Technologies, USA) using GoTaq qPCR Master Mix (Promega, USA) according to the manufacturer's instructions. Threshold cycle (Ct) values were normalized to GAPDH expression, and relative expression levels were calculated using the $2^{-\Delta\Delta Ct}$ method.[45] All primers for human and rat samples were commercially synthesized by BGI (Table S2).

Intracellular ROS concentration assay

Intracellular ROS levels were measured using the CM-H2DCFDA indicator (Invitrogen) according to the manufacturer's instructions. CCC-HEH-2 cells were seeded in 6-well plates at 1.5×10^5 cells per well and cultured for 24 h before hypoxic treatment. After H/R and siRNA intervention, the cells were washed with pre-warmed phosphate-buffered saline, trypsinized, and incubated with the

CM-H2DCFDA indicator for 30 min at 37°C. ROS levels were analyzed using a BD FACSAria III flow cytometer and quantified as median fluorescence intensity (MFI) with FlowJo software.

Intracellular calcium concentrations assay

Cells were incubated with the Fluo-4 AM indicator (Invitrogen) at 37°C for 30 min. Fluorescence intensity was measured with a BD FACSAria III flow cytometer, using excitation at 488 nm and emission at 530 nm. Calcium levels were quantified as MFI with FlowJo software.

Mitochondrial membrane potential assay

Cells were stained with MitoProbe JC-1 assay indicator (Invitrogen), and fluorescence intensities were assessed with a BD FACSAria III flow cytometer at Ex 488 nm/Em 530 nm and Ex 550 nm/Em 600 nm. In healthy cells, JC-1 aggregates in the mitochondria, emitting red fluorescence. In injured cells with compromised membrane potential, JC-1 remains in its monomeric form, emitting green fluorescence. Mitochondrial membrane potential ($\Delta\Psi m$) was quantified by calculating the ratio of red to green fluorescence intensities.

Preparation of HKP-siMIAT nanoparticles

The branched histidine-lysine peptide (HKP) nanoparticle platform (H3K4b variant; Sirnaomics Inc.) was employed for siRNA delivery via electrostatic self-assembly. HKP (2.4 mg) was dissolved in 10 mL RNase-free water and equilibrated at 4°C for 12 h. Subsequently, an equal volume of siRNA solution (80 ng/μL in RNase-free water) was added to the HKP solution. The mixture was incubated at 25°C for 30 min to facilitate nanoparticle formation.

Animal care and MI/R model establishment

Male Sprague-Dawley rats (approximately 220 g) were obtained from Zhuhai Baishitong Biotechnology Co., Ltd. They were housed under a 12-h light/dark cycle with access to tap water and standard chow. All experimental procedures were approved by the ethics committee of the Zhongshan Development Zone Laboratory Animal Center, Guangzhou Analysis and Testing Center, China.

Rats were anesthetized using an MIDMARK anesthesia machine connected to a ventilator, and electrocardiogram (ECG) monitoring was performed before and after the procedure. The chest cavity was accessed through the 3rd or 4th intercostal space, and the heart was exteriorized. The left anterior descending coronary artery (LAD) was ligated 2–3 mm below the left atrial appendage using a 4-0 suture needle. Reperfusion was achieved by releasing the ligature 1 hour after ligation. The sham group underwent the same procedure without LAD ligation.

The rats were randomly assigned into six groups (n = 9–20/group) including sham, MI/R + HKP (serving as a negative control), MI/R + siRNA (7, 14, or 28 nmol/kg encapsulated in HKP nanoparticles), and MI/R + metoprolol (7,505.6 nmol/kg, serving as an active comparator to provide a published reference magnitude in the same rodent MI/R context). All siRNA and placebo were administered intravenously immediately after reperfusion, while metoprolol was administered intragastrically. The animals were sacrificed 16 h post reperfusion, and the hearts were collected for subsequent drug efficacy evaluation.

Myocardial infarct size analysis

The excised hearts were sliced into ~2 mm sections and incubated in a 2% solution of 2,3,5-triphenyl tetrazolium chloride (TTC) at 37°C for 30 min in the dark. This acute-phase assessment (<24 h) is validated by TTC-LGE-MRI correlation studies.[46–48] After staining, the infarcted area appears pale white, while the viable area stains red. The infarct-to-area left ventricular ratio was quantified using ImageJ software.

Histopathological analysis

Heart tissues were fixed in 10% formaldehyde, embedded in paraffin, and sectioned into ~3 μm thick slices. The sections were stained with hematoxylin and eosin (H&E) to assess histopathological changes. Quantitative analysis was conducted using ImageJ software.

Echocardiography

Following isoflurane anesthesia, echocardiography was performed in rats using a VINNO 6LAB ultrasound system (VINNO Technology) with an 18-MHz transducer. M-mode images were captured by identifying the interventricular septum and left ventricular posterior wall. The system automatically calculated left ventricular fractional shortening (FS%) and ejection fraction (EF%).

Markers of myocardial injury assay

Blood samples were collected from the rat abdominal aorta after reperfusion. The samples were centrifuged at 1,000 × g for 20 min to isolate serum. cTnI levels in the serum were determined using a rat-specific ELISA kit (Elabscience), following the manufacturer's protocol. Serum CK-MB and LDH activities were determined with standard kits (Changchun Huili) on a fully automated biochemistry analyzer following the manufacturer's protocols.

RNA-seq analysis

RNA-seq was performed on the BGISEQ-500 platform. Low-quality reads, adapters, and short sequences were removed from the raw data to generate clean data. Clean reads were aligned to the reference genome using HISAT2, and mapping efficiency and gene expression distributions were calculated. Sample reproducibility and specificity were validated through correlation analysis and principal-component analysis. Differential expression analysis identified significantly altered genes ($|\log2 \text{ fold change}| \geq 1$, FDR < 0.05), which were further analyzed for pathway enrichment to gain insights into the molecular mechanisms underlying the observed phenotypes.

Determination of cAMP levels by ELISA

Supernatants from cardiac tissue homogenates were collected by centrifugation at 5,000 × g for 10 min. cAMP levels in the

supernatants were measured using a cAMP ELISA kit (Elabscience) according to the manufacturer's protocol.

Statistical analysis

Data are presented as means ± SDs. Statistical analysis was performed using GraphPad Prism (version 8.0). Differences between groups were assessed using one-way ANOVA, with statistical significance set at $p < 0.05$.

DATA AND CODE AVAILABILITY

The data that support the findings of this study are available within the article and its supplemental information.

ACKNOWLEDGMENTS

This work was financially funded by Macao Science and Technology Development Fund, Macau SAR (file no. 0001/2023/AKP to Z.-H.J. and no. 0012/2022/AGJ to T.-M.Y.). This work was funded by Department of Science and Technology of Guangdong Province; Macao Science and Technology Development Fund (Project No.: no. 2025B1212040001 and no. 0002/2025/COP). We are grateful to Dr. Yang Lu (Sirnaomics) for the gift of HKP for RNA delivery *in vivo* and his technical advice. This work was partially supported by Ruina (Zhuhai Hengqin) Biothechnology.

AUTHOR CONTRIBUTIONS

Z.-H.J., T.-M.Y., and X.-R.M. conceived the study. X.-R.M. and T.-M.Y. designed the experiments. X.-R.M., T.-M.Y., and Y.P. conducted the experiments. X.-R.M. and T.-M.Y analyzed the data. X.-R.M., T.-M.Y., and Z.-H.J. drafted the manuscript. All of the authors have reviewed and approved the manuscript.

DECLARATION OF INTERESTS

The authors declare no competing interests.

SUPPLEMENTAL INFORMATION

Supplemental information can be found online at https://doi.org/10.1016/j.omtn.2025.102747.

REFERENCES

1. An, Y.L., Fu, P., Li, Z.Z., Zhang, Y.L., Lin, Q.Y., and Zhang, B. (2024). Nicorandil activates lkb1-ampk signaling pathway to ameliorate cardiac remodeling after myocardial ischemia/reperfusion injury by upregulating mt2. Eur. Heart J. *45*, ehae666.1517. https://doi.org/10.1093/eurheartj/ehae666.1517.
2. Yellon, D.M., and Hausenloy, D.J. (2007). Myocardial reperfusion injury. N. Engl. J. Med. *357*, 1121–1135. https://doi.org/10.1056/NEJMra071667.
3. World Health Organization (2024). The top 10 causes of death. https://www.who.int/news-room/fact-sheets/detail/the-top-10-causes-of-death.
4. Bangalore, S., Maron, D.J., Stone, G.W., and Hochman, J.S. (2020). Routine revascularization versus initial medical therapy for stable ischemic heart disease: a systematic review and meta-analysis of randomized trials. Circulation *142*, 841–857. https://doi.org/10.1161/CIRCULATIONAHA.120.048194.
5. Neumann, F.-J., Sousa-Uva, M., Ahlsson, A., Alfonso, F., Banning, A.P., Benedetto, U., Byrne, R.A., Collet, J.-P., Falk, V., Head, S.J., et al. (2019). 2018 ESC/EACTS Guidelines on myocardial revascularization. Eur. Heart J. *40*, 87–165. https://doi.org/10.1093/eurheartj/ehy394.
6. Hausenloy, D.J., Chilian, W., Crea, F., Davidson, S.M., Ferdinandy, P., Garcia-Dorado, D., van Royen, N., Schulz, R., and Heusch, G. (2019). The coronary circulation in acute myocardial ischaemia/reperfusion injury: a target for cardioprotection. Cardiovasc. Res. *115*, 1143–1155. https://doi.org/10.1093/cvr/cvy286.
7. Takahashi, J., Nihei, T., Takagi, Y., Miyata, S., Odaka, Y., Tsunoda, R., Seki, A., Sumiyoshi, T., Matsui, M., Goto, T., et al. (2015). Prognostic impact of chronic nitrate therapy in patients with vasospastic angina: multicentre registry study of the Japanese coronary spasm association. Eur. Heart J. *36*, 228–237. https://doi.org/10.1093/eurheartj/ehu313.
8. Held, C., Åsenblad, N., Bassand, J.P., Becker, R.C., Cannon, C.P., Claeys, M.J., Harrington, R.A., Horrow, J., Husted, S., James, S.K., et al. (2011). Ticagrelor versus clopidogrel in patients with acute coronary syndromes undergoing coronary artery bypass surgery: results from the PLATO (Platelet Inhibition and Patient Outcomes) trial. J. Am. Coll. Cardiol. *57*, 672–684. https://doi.org/10.1016/j.jacc.2010.10.029.
9. Barangi, S., Hayes, A.W., Reiter, R., and Karimi, G. (2019). The therapeutic role of long non-coding RNAs in human diseases: a focus on the recent insights into autophagy. Pharmacol. Res. *142*, 22–29. https://doi.org/10.1016/j.phrs.2019.02.010.
10. Wang, Y., and Sun, X. (2020). The functions of LncRNA in the heart. Diabetes Res. Clin. Pract. *168*, 108249. https://doi.org/10.1016/j.diabres.2020.108249.
11. Tian, Y., Gao, Z., Liu, W., Li, J., Jiang, X., and Xin, Y. (2022). Unveiling the vital role of long non-coding RNAs in cardiac oxidative stress, cell death, and fibrosis in diabetic cardiomyopathy. Antioxidants *11*, 2391. https://doi.org/10.3390/antiox11122391.
12. Zhao, Z., Sun, W., Guo, Z., Liu, B., Yu, H., and Zhang, J. (2021). Long noncoding RNAs in myocardial ischemia-reperfusion injury. Oxid. Med. Cell. Longev. *2021*, 8889123. https://doi.org/10.1155/2021/8889123.
13. Yang, C., Zhang, Y., and Yang, B. (2021). MIAT, a potent CVD-promoting lncRNA. Cell. Mol. Life Sci. *79*, 43. https://doi.org/10.1007/s00018-021-04046-8.
14. Ishii, N., Ozaki, K., Sato, H., Mizuno, H., Saito, S., Takahashi, A., Miyamoto, Y., Ikegawa, S., Kamatani, N., Hori, M., et al. (2006). Identification of a novel non-coding RNA, MIAT, that confers risk of myocardial infarction. J. Hum. Genet. *51*, 1087–1099. https://doi.org/10.1007/s10038-006-0070-9.
15. Chen, L., Zhang, D., Yu, L., and Dong, H. (2019). Targeting MIAT reduces apoptosis of cardiomyocytes after ischemia/reperfusion injury. Bioengineered *10*, 121–132. https://doi.org/10.1080/21655979.2019.1605812.
16. Qu, X., Du, Y., Shu, Y., Gao, M., Sun, F., Luo, S., Yang, T., Zhan, L., Yuan, Y., Chu, W., et al. (2017). MIAT is a pro-fibrotic long non-coding RNA governing cardiac fibrosis in post-infarct myocardium. Sci. Rep. *7*, 42657. https://doi.org/10.1038/srep42657.
17. Gupta, P. (2006). RNA interference–gene silencing by double-stranded RNA: The 2006 Nobel Prize for Physiology or Medicine. Curr. Sci. *91*, 1443.
18. Fire, A., Xu, S., Montgomery, M.K., Kostas, S.A., Driver, S.E., and Mello, C.C. (1998). Potent and specific genetic interference by double-stranded RNA in Caenorhabditis elegans. Nature *391*, 806–811. https://doi.org/10.1038/35888.
19. Zimmermann, T.S., Karsten, V., Chan, A., Chiesa, J., Boyce, M., Bettencourt, B.R., Hutabarat, R., Nochur, S., Vaishnaw, A., and Gollob, J. (2017). Clinical proof of concept for a novel hepatocyte-targeting GalNAc-siRNA conjugate. Mol. Ther. *25*, 71–78. https://doi.org/10.1016/j.ymthe.2016.10.019.
20. Egli, M., and Manoharan, M. (2023). Chemistry, structure and function of approved oligonucleotide therapeutics. Nucleic Acids Res. *51*, 2529–2573. https://doi.org/10.1093/nar/gkad067.
21. Trajanoska, K., Bhérer, C., Taliun, D., Zhou, S., Richards, J.B., and Mooser, V. (2023). From target discovery to clinical drug development with human genetics. Nature *620*, 737–745. https://doi.org/10.1038/s41586-023-06388-8.
22. Hu, K., Yan, T.-M., Cao, K.-Y., Li, F., Ma, X.-R., Lai, Q., Liu, J.-C., Pan, Y., Kou, J.-P., and Jiang, Z.-H. (2022). A tRNA-derived fragment of ginseng protects heart against ischemia/reperfusion injury via targeting the lncRNA MIAT/VEGFA pathway. Mol. Ther. Nucleic Acids *29*, 672–688. https://doi.org/10.1016/j.omtn.2022.08.014.
23. Brubaker, D.K., and Lauffenburger, D.A. (2020). Translating preclinical models to humans. Science *367*, 742–743. https://doi.org/10.1126/science.aay8086.
24. Huang, F., Du, J., Liang, Z., Xu, Z., Xu, J., Zhao, Y., Lin, Y., Mei, S., He, Q., Zhu, J., et al. (2019). Large-scale analysis of small RNAs derived from traditional Chinese herbs in human tissues. Sci. China Life Sci. *62*, 321–332. https://doi.org/10.1007/s11427-018-9323-5.
25. Kang, P.M., Haunstetter, A., Aoki, H., Usheva, A., and Izumo, S. (2000). Morphological and molecular characterization of adult cardiomyocyte apoptosis during hypoxia and reoxygenation. Circ. Res. *87*, 118–125. https://doi.org/10.1161/01.RES.87.2.118.
26. Xu, Y., Xing, Y., Xu, Y., Huang, C., Bao, H., Hong, K., and Cheng, X. (2016). Pim-2 protects H9c2 cardiomyocytes from hypoxia/reoxygenation-induced apoptosis via

downregulation of Bim expression. Environ. Toxicol. Pharmacol. *48*, 94–102. https://doi.org/10.1016/j.etap.2016.10.011.

27. Veldhoen, S., Laufer, S.D., Trampe, A., and Restle, T. (2006). Cellular delivery of small interfering RNA by a non-covalently attached cell-penetrating peptide: quantitative analysis of uptake and biological effect. Nucleic Acids Res. *34*, 6561–6573. https://doi.org/10.1093/nar/gkl941.

28. Vaidyanathan, S., Orr, B.G., and Banaszak Holl, M.M. (2016). Role of Cell Membrane–Vector Interactions in Successful Gene Delivery. Acc. Chem. Res. *49*, 1486–1493. https://doi.org/10.1021/acs.accounts.6b00200.

29. Guo, H., Xu, X., Zhang, J., Du, Y., Yang, X., He, Z., Zhao, L., Liang, T., and Guo, L. (2024). The Pivotal Role of Preclinical Animal Models in Anti-Cancer Drug Discovery and Personalized Cancer Therapy Strategies. Pharmaceuticals *17*, 1048. https://doi.org/10.3390/ph17081048.

30. Qi, Y., Wu, H., Mai, C., Lin, H., Shen, J., Zhang, X., Gao, Y., Mao, Y., and Xie, X. (2020). LncRNA-MIAT-mediated miR-214-3p silencing is responsible for IL-17 production and cardiac fibrosis in diabetic cardiomyopathy. Front. Cell Dev. Biol. *8*, 243. https://doi.org/10.3389/fcell.2020.00243.

31. Shen, S., Liao, Q., Gu, L., Zhu, Y., Liu, Y., Zhang, X., Zhang, J., Shi, Q., Sun, Y., Wang, J., et al. (2024). G protein-coupled receptor-biased signaling: potential drug discovery to facilitate treatment of metabolic diseases. Acta Mater. Med. *3*, 31–45. https://doi.org/10.15212/AMM-2023-0041.

32. Beheshti, S., Ershadi, S., Zamani, F., Azimzadeh, M., and Wesal, M.W. (2023). Differential impact of a ghrelin receptor antagonist or inverse agonist in the electrical kindling model of epilepsy. Epilepsy Res. *197*, 107234. https://doi.org/10.1016/j.eplepsyres.2023.107234.

33. Zhang, Y., Wang, X.-L., Zhao, J., Wang, Y.-J., Lau, W.B., Yuan, Y.-X., Gao, E.-H., Koch, W.J., and Ma, X.-L. (2013). Adiponectin inhibits oxidative/nitrative stress during myocardial ischemia and reperfusion via PKA signaling. Am. J. Physiol. Endocrinol. Metab. *305*, E1436–E1443. https://doi.org/10.1152/ajpendo.00445.2013.

34. Lu, W.-W., Jia, L.-X., Ni, X.-Q., Zhao, L., Chang, J.-R., Zhang, J.-S., Hou, Y.-L., Zhu, Y., Guan, Y.-F., Yu, Y.-R., et al. (2016). Intermedin1− 53 Attenuates Abdominal Aortic Aneurysm by Inhibiting Oxidative Stress. Arterioscler. Thromb. Vasc. Biol. *36*, 2176–2190. https://doi.org/10.1161/ATVBAHA.116.307825.

35. Byrne, R.A., Rossello, X., Coughlan, J.J., Barbato, E., Berry, C., Chieffo, A., Claeys, M.J., Dan, G.-A., Dweck, M.R., Galbraith, M., et al. (2024). 2023 ESC Guidelines for the management of acute coronary syndromes: Developed by the task force on the management of acute coronary syndromes of the European Society of Cardiology (ESC). Eur. Heart J. Acute Cardiovasc. Care *13*, 55–161. https://doi.org/10.1093/ehjacc/zuad107.

36. Divakaran, S., and Loscalzo, J. (2017). The Role of Nitroglycerin and Other Nitrogen Oxides in Cardiovascular Therapeutics. J. Am. Coll. Cardiol. *70*, 2393–2410. https://doi.org/10.1016/j.jacc.2017.09.1064.

37. Pfeffer, M.A., Braunwald, E., Moyé, L.A., Basta, L., Brown, E.J., Cuddy, T.E., Davis, B.R., Geltman, E.M., Goldman, S., Flaker, G.C., et al. (1992). Effect of Captopril on Mortality and Morbidity in Patients with Left Ventricular Dysfunction after Myocardial Infarction. N. Engl. J. Med. *327*, 669–677. https://doi.org/10.1056/NEJM199209033271001.

38. Qu, X., Du, Y., Shu, Y., Gao, M., Sun, F., Luo, S., Yang, T., Zhan, L., Yuan, Y., Chu, W., et al. (2017). MIAT Is a Pro-fibrotic Long Non-coding RNA Governing Cardiac Fibrosis in Post-infarct Myocardium. Sci. Rep. *7*, 42657. https://doi.org/10.1038/srep42657.

39. Ibanez, B., Macaya, C., Sánchez-Brunete, V., Pizarro, G., Fernández-Friera, L., Mateos, A., Fernández-Ortiz, A., García-Ruiz, J.M., García-Álvarez, A., Iñiguez, A., et al. (2013). Effect of Early Metoprolol on Infarct Size in ST-Segment–Elevation Myocardial Infarction Patients Undergoing Primary Percutaneous Coronary Intervention. Circulation *128*, 1495–1503. https://doi.org/10.1161/CIRCULATIONAHA.113.003653.

40. Roolvink, V., Ibáñez, B., Ottervanger, J.P., Pizarro, G., van Royen, N., Mateos, A., Dambrink, J.-H.E., Escalera, N., Lipsic, E., Albarran, A., et al. (2016). Early Intravenous Beta-Blockers in Patients With ST-Segment Elevation Myocardial Infarction Before Primary Percutaneous Coronary Intervention. J. Am. Coll. Cardiol. *67*, 2705–2715. https://doi.org/10.1016/j.jacc.2016.03.522.

41. Zhou, H., Ma, Q., Zhu, P., Ren, J., Reiter, R.J., and Chen, Y. (2018). Protective role of melatonin in cardiac ischemia-reperfusion injury: From pathogenesis to targeted therapy. J. Pineal Res. *64*, e12471. https://doi.org/10.1111/jpi.12471.

42. Yu, H., Kalogeris, T., and Korthuis, R.J. (2019). Reactive species-induced microvascular dysfunction in ischemia/reperfusion. Free Radic. Biol. Med. *135*, 182–197. https://doi.org/10.1016/j.freeradbiomed.2019.02.031.

43. Drab, M., Verkade, P., Elger, M., Kasper, M., Lohn, M., Lauterbach, B., Menne, J., Lindschau, C., Mende, F., Luft, F.C., et al. (2001). Loss of Caveolae, Vascular Dysfunction, and Pulmonary Defects in Caveolin-1 Gene-Disrupted Mice. Science *293*, 2449–2452. https://doi.org/10.1126/science.1062688.

44. Bucci, M., Gratton, J.-P., Rudic, R.D., Acevedo, L., Roviezzo, F., Cirino, G., and Sessa, W.C. (2000). In vivo delivery of the caveolin-1 scaffolding domain inhibits nitric oxide synthesis and reduces inflammation. Nat. Med. *6*, 1362–1367. https://doi.org/10.1038/82176.

45. Schmittgen, T.D., and Livak, K.J. (2008). Analyzing real-time PCR data by the comparative CT method. Nat. Protoc. *3*, 1101–1108. https://doi.org/10.1038/nprot.2008.73.

46. Xia, R., Lu, X., Zhang, B., Wang, Y., Liao, J., Zheng, J., and Gao, F. (2015). Early reperfusion can reduce infarction size but not salvaged myocardial size in acute myocardial infarction rats. JACC. Cardiovasc. Imaging *8*, 616–617. https://doi.org/10.1016/j.jcmg.2014.07.023.

47. Xu, L.-j., Chen, R.-c., Ma, X.-y., Zhu, Y., Sun, G.-b., and Sun, X.-b. (2020). Scutellarin protects against myocardial ischemia-reperfusion injury by suppressing NLRP3 inflammasome activation. Phytomedicine *68*, 153169. https://doi.org/10.1016/j.phymed.2020.153169.

48. Yang, W., Wang, Y., Fu, C., Li, C., Feng, F., Li, H., Tan, L., Qu, H., Hui, H., Wang, J., et al. (2024). Quantitative visualization of myocardial ischemia-reperfusion-induced cardiac lesions via ferroptosis magnetic particle imaging. Theranostics *14*, 1081–1097. https://doi.org/10.7150/thno.89190.

OMTN, Volume *36*

Supplemental information

Optimization of siRNA therapeutics targeting MIAT for cardioprotection in myocardial ischemia/reperfusion injury

Xiao-Rong Ma, Tong-Meng Yan, Yu Pan, and Zhi-Hong Jiang

Supplemental information

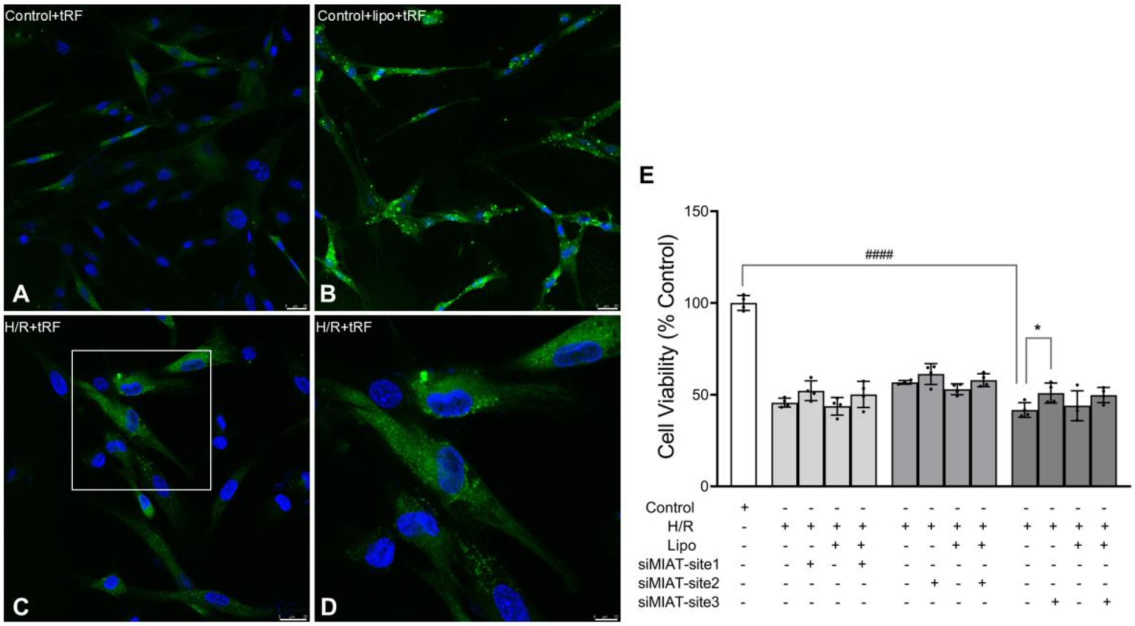

Figure S1. tRF efficiently entered H/R-injured cells without the use of transfection reagents.

Nuclei were stained with Hoechst 33342, appearing blue. (A) The image shows normal cells treated with 100 nM Cy5-labeled HC83 without a transfection reagent. (B) The fluorescence image shows normal cells treated with 100 nM HC83, utilizing Lipofectamine RNAiMAX as the transfection reagent. (C) The image of H/R-injured cells treated with 100 nM Cy5-labeled HC83 without using transfection reagents. Images (A) and (B) were taken with a 20 × objective lens, with scale bars at 25 μm. (D) Offers a magnified view of the area marked in (C), with a 10 μm scale bar, emphasizing the enhanced cellular uptake of HC83 in H/R-injured cells. (E) The comparison of the effects of siMIAT-site1, siMIAT-site2, and siMIAT-site3 on the viability of H/R-induced injured cells at a concentration of 100 nM, both with or without Lipofectamine, reveals that siRNAs demonstrate slightly greater protective activity when the transfection reagent is not used. Data are presented as means ± SDs, n=4. P values were calculated using One-way ANOVA. ####$p < 0.0001$ vs. Control; ∗$p < 0.05$ vs. H/R.

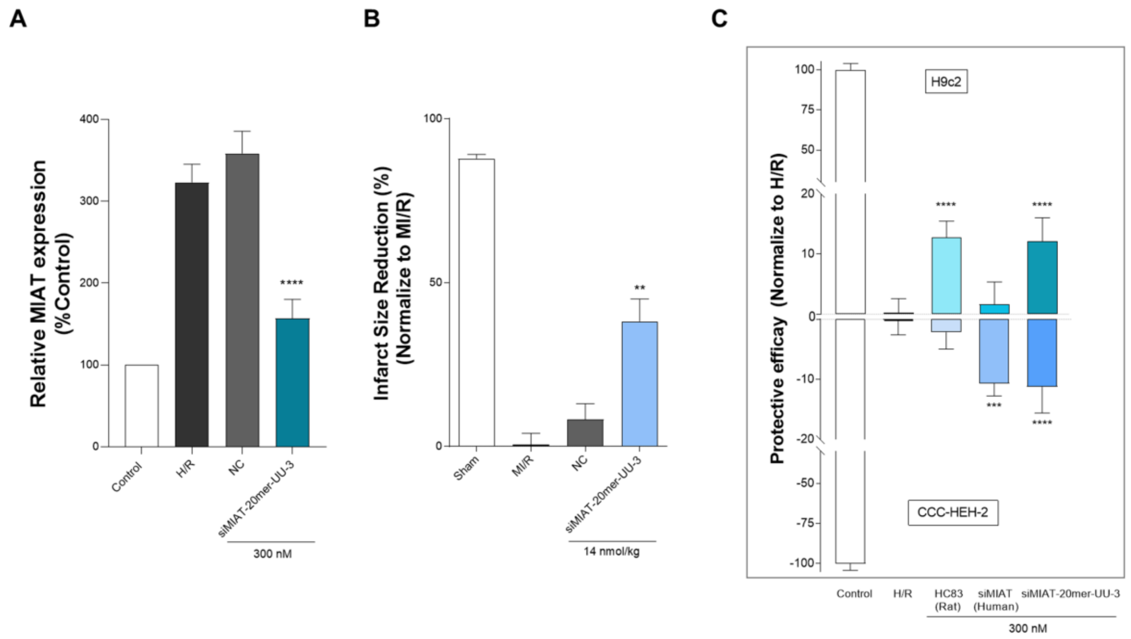

Figure S2. Effects of siRNA treatments on MIAT expression and cardiomyocytes protection.

(A) MIAT expression in H9c2 cardiomyocytes under H/R injury treated with siNC or siMIAT-20mer-UU-3. (B) Infarct size in MI/R model rats treated with siNC or siMIAT-20mer-UU-3 (14 ng/kg). (C) Cell viability in H9c2 and CCC-HEH-2 cardiomyocytes treated with: HC83 (rat-specific), siMIAT-human (human-specific), or siMIAT-20mer-UU-3. Data are presented as means ± SDs. P values were calculated using One-way ANOVA. **p < 0.01 vs. MI/R, ***p < 0.001, ****p<0.0001 vs. H/R.

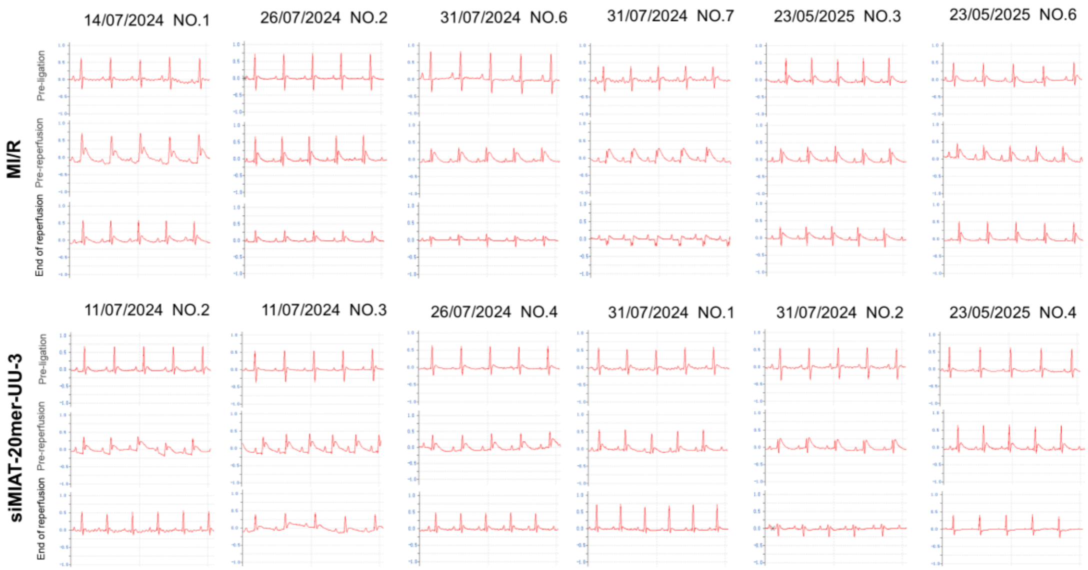

Figure S3. ECG recordings from MI/R and siMIAT-20mer-UU-3 treatment groups.

Comparison of ECG recordings between MI/R and siMIAT-20mer-UU-3 treatment groups across different time points.

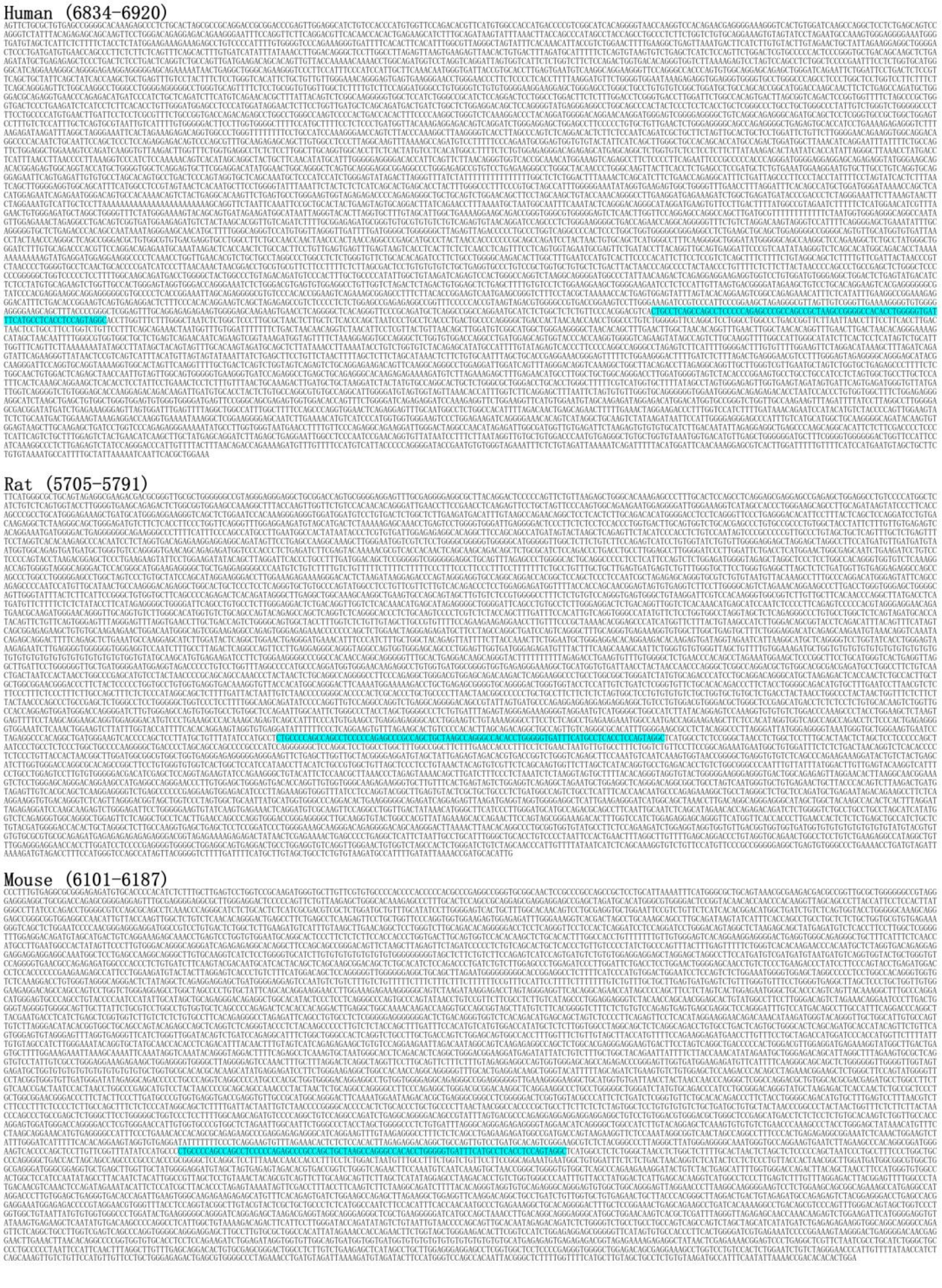

Figure S4. The complete FASTA sequences and homologous regions of lncRNA MIAT from three species.

The blue-highlighted portions indicate the homologous regions of the three sequences.

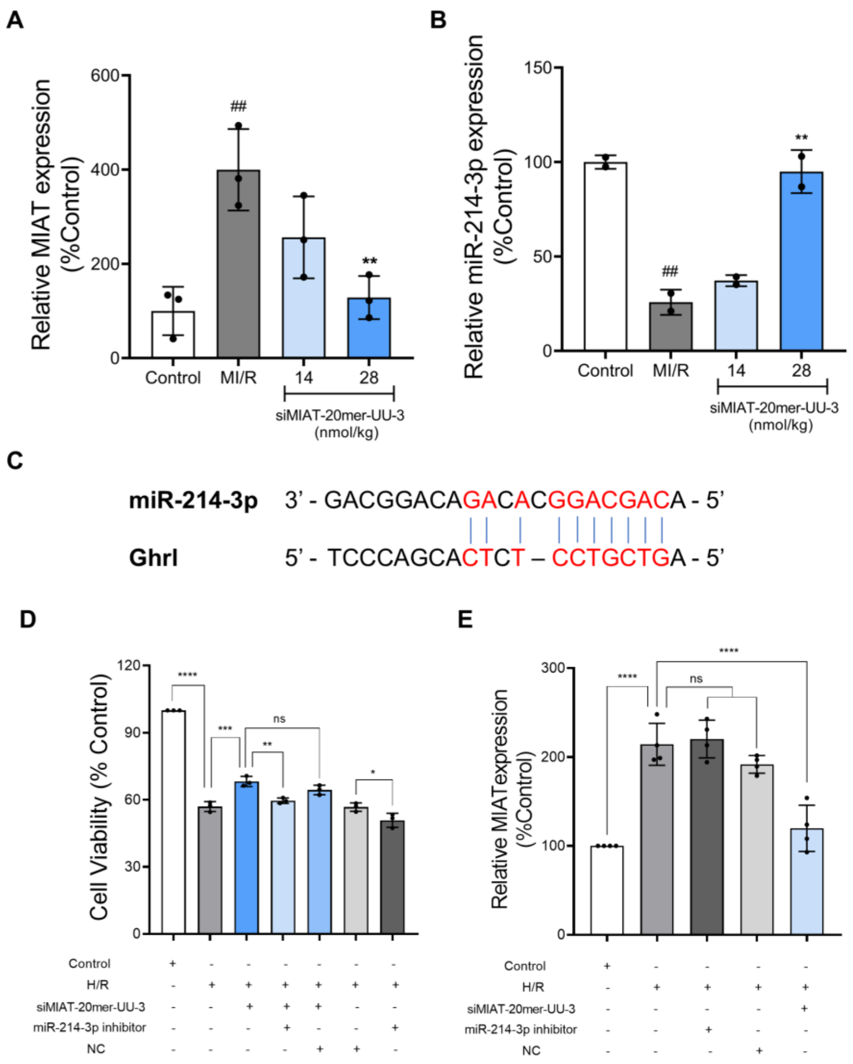

Figure S5. siMIAT-20mer-UU-3 treatment reversed MIAT upregulation and affected miR-214-3p expression, which may potentially target Ghrl.

(A) Relative MIAT expression levels (% of control) were measured in different groups: control, MI/R, and siMIAT-20mer-UU-3 treated at 14 and 28 nmol/kg dosages. (B) Relative miR-214-3p expression levels (% of control) were assessed. P values were calculated using One-way ANOVA. ##p < 0.01 vs. Control; **p< 0.01 vs. MI/R. (C) Sequence alignment predicted the potential binding site between miR-

214-3p and its target gene, Ghrl. The PCR results from the same batch of samples used for RNA sequencing showed that treatment with siMIAT-20mer-UU-3 reversed the upregulation of MIAT observed in the model group. It suggested that MIAT may act as a molecular sponge to regulate miR-214-3p, which is predicted to target the Ghrl gene based on the miRanda database. (D) Effects of siMIAT-20mer-UU-3 and miR-214-3p inhibitor on cell viability in H9c2 cells subjected to H/R conditions. Data are presented as means ± SDs from 3 independent experiments. (E) MIAT RNA expression was measured by quantitative PCR in H/R-injured H9c2 cells treated with: Control, H/R, miR-214-3p inhibitor, negative control inhibitor and siMIAT-20mer-UU-3. Data are presented as means ± SDs from 4 independent experiments. P values were calculated using one-way ANOVA. $*p < 0.05$, $**p < 0.01$, $***p < 0.001$, $****p < 0.0001$.

Table S1. Information of twenty optimized siRNAs.

siRNA Name	Passenger strand (5'-3')	Length (mer)	Guide strand (5'-3')	Length (mer)
siMIAT-19mer-1	UUUCAUGCCUCACCUCCAG	19	CUGGAGGUGAGGCAUGAAA	19
siMIAT-19mer-2	UUCAUGCCUCACCUCCAGU		ACUGGAGGUGAGGCAUGAA	
siMIAT-19mer-3	UCAUGCCUCACCUCCAGUA		UACUGGAGGUGAGGCAUGA	
siMIAT-19mer-4	CAUGCCUCACCUCCAGUAG		CUACUGGAGGUGAGGCAUG	
siMIAT-19mer-5	AUGCCUCACCUCCAGUAGG		CCUACUGGAGGUGAGGCAU	
siMIAT-19mer-UU-1	UUUCAUGCCUCACCUCCAGUU	21	CUGGAGGUGAGGCAUGAAAUU	21
siMIAT-19mer-UU-2	UUCAUGCCUCACCUCCAGUUU		ACUGGAGGUGAGGCAUGAAUU	
siMIAT-19mer-UU-3	UCAUGCCUCACCUCCAGUAUU		UACUGGAGGUGAGGCAUGAUU	
siMIAT-19mer-UU-4	CAUGCCUCACCUCCAGUAGUU		CUACUGGAGGUGAGGCAUGUU	
siMIAT-19mer-UU-5	AUGCCUCACCUCCAGUAGGUU		CCUACUGGAGGUGAGGCAUUU	
siMIAT-20mer-1	AUUUCAUGCCUCACCUCCAG	20	CUGGAGGUGAGGCAUGAAAU	20
siMIAT-20mer-2	UUUCAUGCCUCACCUCCAGU		ACUGGAGGUGAGGCAUGAAA	
siMIAT-20mer-3	UUCAUGCCUCACCUCCAGUA		UACUGGAGGUGAGGCAUGAA	
siMIAT-20mer-4	UCAUGCCUCACCUCCAGUAG		CUACUGGAGGUGAGGCAUGA	
siMIAT-20mer-5	CAUGCCUCACCUCCAGUAGG		CCUACUGGAGGUGAGGCAUG	
siMIAT-20mer-UU-1	AUUUCAUGCCUCACCUCCAGUU	22	CUGGAGGUGAGGCAUGAAAUUU	22
siMIAT-20mer-UU-2	UUUCAUGCCUCACCUCCAGUUU		ACUGGAGGUGAGGCAUGAAAUU	
siMIAT-20mer-UU-3	UUCAUGCCUCACCUCCAGUAUU		UACUGGAGGUGAGGCAUGAAUU	
siMIAT-20mer-UU-4	UCAUGCCUCACCUCCAGUAGUU		CUACUGGAGGUGAGGCAUGAUU	
siMIAT-20mer-UU-5	CAUGCCUCACCUCCAGUAGGUU		CCUACUGGAGGUGAGGCAUGUU	

Table S2. Primers for quantitative real-time PCR.

Gene name		Sequence of oligonucleotides
H-GAPDH	F	GTCTCCTCTGACTTCAACAGCG
	R	ACCACCCTGTTGCTGTAGCCAA
H-MIAT	F	GGACGTTCACAACCACACTG
	R	TCCCACTTTGGCATTCTAGG
R-GAPDH	F	TGCCCAGAACATCATCCCT
	R	GGTCCTCAGTGTAGCCCAAG
R-MIAT	F	ACCAGCAACGGAGTAGTGTG
	R	CACAGCCCCGGAATGAAGAGT
R-U6	RT	AACGCTTCACGAATTTGCGT
	F	CTCGCTTCGGCAGCACAT
	R	AACGCTTCACGAATTTGCGT
R-miR-214-3p	RT	GTCGTATCCAGTGCAGGGTCCGAGGTATTCGCACTGGATACGACCTGCCT
	F	GCGGCGGACAGCAGGCACAGAC
	R	ATCCAGTGCAGGGTCCGAGG

Abbreviations: RT = reverse transcription; F = forward; R = reverse.
